# Pervasive sex-dependent effects in the genetic architecture of starvation resistance in *Drosophila melanogaster*

**DOI:** 10.1126/sciadv.adt3560

**Published:** 2025-09-26

**Authors:** Junhao Chen, Chenlu Liu, Weixuan Li, Andrew G. Clark, Zhijuan Wang, Jian Lu

**Affiliations:** ^1^ State Key Laboratory of Gene Function and Modulation Research, Center for Bioinformatics, School of Life Sciences, Peking University, Beijing, China.; ^2^ Department of Molecular Biology and Genetics, Cornell University, Ithaca, NY 14853, USA.; ^3^ Southwest United Graduate School, Kunming 650092, China.; ^4^ Beijing Advanced Center of RNA Biology (BEACON), Peking University, Beijing 100871, China.

## Abstract

Genetic variants can have sex-specific, sex-biased, or sexually antagonistic fitness effects, yet their roles in fitness-related traits remain unclear. Using pooled phenotype sorting and sequencing of male *Drosophila melanogaster* from natural populations, we identified starvation resistance–associated variants, many in regulatory regions or altering protein sequences. RNA interference experiments showed that 85.7% (66 of 77) of the candidate genes with nonlethal knockdown effects influenced starvation resistance. Of these, 49 had sex-dependent effects, including 12 with sexually antagonistic effects—all increasing resistance in females but decreasing it in males. These patterns were not explained by sex-biased expression or knockdown efficiency. Analysis of the *Lnk* gene revealed that both nonsynonymous mutations and expression changes had sex-dependent effects. Our findings indicate that polygenic architecture, sex-dependent effects, and pleiotropy jointly shape evolutionary outcomes and that some variants maintained by these forces may enable rapid responses to environmental change.

## Teaser

Genetic architecture of starvation resistance in *Drosophila*: ~70% candidate genes exhibit sex-dependent effects.

## INTRODUCTION

A genetic variant can have different fitness consequences in males and females because of differences in physiology, hormonal environment, or other factors, leading to sex-dependent gene effects (*1*). Sexual conflict, or sexual antagonism, occurs when a genetic variant has opposite fitness effects in males and females, meaning that an allele beneficial to one sex is detrimental to the other (*2*–*10*).

Environmental changes can differentially affect male and female fitness, given their distinct survival and reproduction strategies (*11*, *12*). While sex-dependent effects might diminish under environmental stress because of aligned selection pressures, the role of genes with sex-biased or antagonistic effects in many fitness-related traits remains debated (*13*–*15*). On the one hand, sexual conflict can prevent sex-specific optimization and result in offspring with suboptimal trait combinations, which may impede the population’s response to selection (*16*, *17*). Conversely, it may maintain fitness-related variation through balancing selection, fostering allele diversity for a rapid response to environmental changes (*18*–*20*). Despite these considerations, identifying the genes responsible for sex-dependent effects and deciphering their impact on fitness-related traits in natural populations remain challenging (*21*).

Studies in *Drosophila melanogaster* have revealed sex-dependent differences in a wide range of polygenic traits, such as lipid profiles, nutrient requirements, and regulation of feeding behavior (*22*, *23*), suggesting the presence of sex-biased or antagonistic effects at the phenotypic level. Laboratory evolution experiments in *Drosophila* have provided direct evidence for sexual conflict by demonstrating negative genetic correlations of fitness between sexes (*24*). Selection for traits like desiccation resistance has shown trade-offs between male and female fitness, further highlighting sexual conflict (*25*). A genome-wide association study identified 230 potentially antagonistic single-nucleotide polymorphism (SNP) clusters in *D. melanogaster* (*18*). Identification of individual genes of large effect often reveals sex differences at the single gene level. Examples of antagonistic genes in *D. melanogaster* include *Apollo* and *Artemis* and their impacts on fertility (*26*), *fezzik* and its effect on starvation resistance (*27*), and *Cyp6g1* and its role in sexual conflict over fitness (*28*). These findings underscore the importance of *D. melanogaster* as a model system for investigating the genetic basis and evolutionary consequences of sex-dependent gene effects.

We investigated sex-dependent gene effects in response to acute starvation in natural *D. melanogaster* populations. Starvation resistance, a trait with substantial genetic diversity, is vital for survival in fluctuating environments (*29*–*35*). However, few identified candidate genes overlap across previous studies or have undergone functional validation. Rapid environmental changes and differences between natural and lab conditions further complicate the identification of genes linked to starvation resistance (*36*, *37*). We identified genetic variants linked to starvation resistance in natural *D. melanogaster* populations using a new method. Using RNA interference (RNAi) knockdown and gene replacement experiments, we identified genetic variants associated with starvation resistance and their sex-dependent effects.

## RESULTS

### PCSS method for identifying starvation resistance variants in *D. melanogaster*

**Fig. 1.**
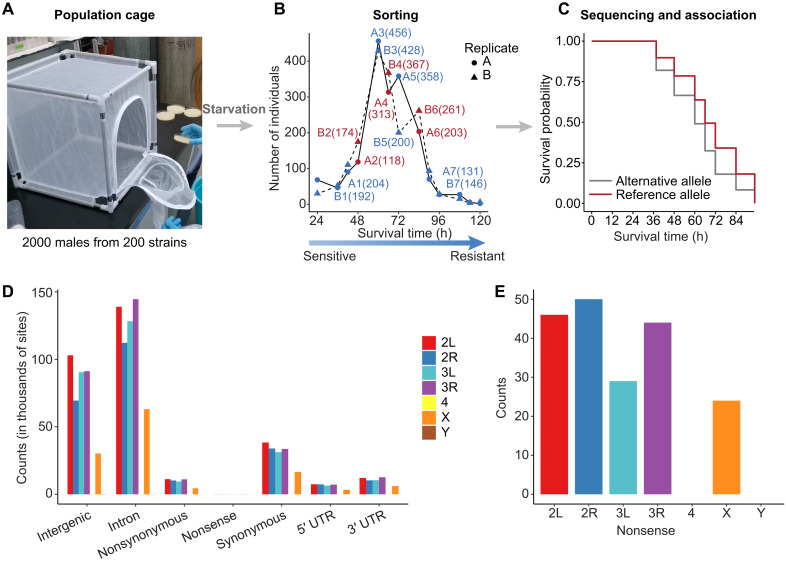
Overview of the PCSS approach for identifying starvation resistance–associated variants in *D. melanogaster*. (**A**) Experimental setup for the population cage, in which 2000 male flies from 200 isofemale strains (10 male adults per strain) collected from 21 diverse locations in China were pooled and subjected to starvation. (**B**) Flies were sorted into seven phenotypic groups on the basis of survival time during starvation. The flies were categorized from most sensitive to most resistant, with two replicates (A and B) used for consistency. The number of individuals in each sample is shown in parentheses. h, hours. (**C**) Illustration of the application of the Cox proportional hazards regression model to one genetic variant to analyze its association with starvation resistance. The survival probabilities of individuals with the reference and alternative alleles are compared. (**D**) Distribution of genetic variants by type (missense, nonsense, synonymous, UTR, intronic, and intergenic), with counts presented for each chromosome. (**E**) Breakdown of the fewer-numbered nonsense variants shown in (D).

We developed a high-throughput and cost-effective method, population cage sorting and sequencing (PCSS), to identify genetic variants associated with starvation resistance in *D. melanogaster* (Fig. 1, A to C). PCSS uses population cages to test up to 2000 individuals per trial, ensuring uniform testing environments for all flies and thereby minimizing batch effects commonly encountered in traditional genome-wide association studies that use individual vials. For this, we pooled 200 isofemale strains (10 male adults from one strain) from 21 diverse locations in China (fig. S1 and table S1) into a population cage and subjected them to starvation. Dead individuals were collected every 6 to 12 hours and grouped into seven samples on the basis of survival time (table S2). The experiments were conducted with two biological replicates (A and B), and the genomic DNA of each pooled sample was sequenced using Pool-seq on the Illumina platform to a depth of ~200×. In parallel, two untreated samples (C1 and C2), each containing around 1000 males (five males from each strain), were sequenced to a depth of ~400× as background references.

High-quality variants were called in C1 and C2 and filtered on the basis of depth and minor allele counts (*38*). We obtained a total of 1,409,919 variants across the two samples, with 1,285,620 variants being polymorphic in both replicates for downstream analysis. These included 587,234 intron SNPs, 384,358 intergenic SNPs, 46,753 missense SNPs, 153,654 synonymous SNPs, 35,867 SNPs in the 5′ untranslated region (5′UTR), 51,571 SNPs in the 3′ untranslated region (3′UTR), and 195 nonsense SNPs (Fig. 1, D and E). Allele frequencies estimated by Pool-seq data of C1 and C2 were highly correlated with those based on whole-genome sequencing of 292 isofemale strains (Pearson’s *r* = 0.990; fig. S2) collected in China (*39*), suggesting that Pool-seq allele frequency estimation is reliable. Linkage disequilibrium decayed rapidly, with *r*^2^ dropping below 0.2 within ~300 bp (base pairs) on autosomes (fig. S3), indicating that there was not sufficient genetic differentiation among the subpopulations to generate spurious sampling linkage disequilibrium. Sequencing results of each pooled sample under starvation recaptured more than 98.4% of the SNPs in the C1 and C2 background references (table S3).

**Fig. 2.**
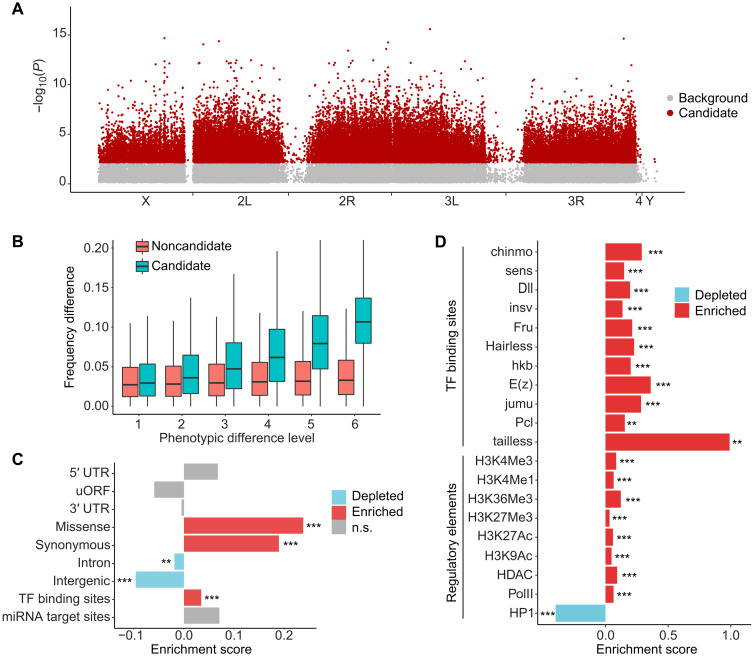
Genetic architecture of starvation resistance–associated variants in *Drosophila melanogaster*. (**A**) Genome-wide distribution of SNPs associated with starvation resistance. The *P* value was calculated using Cox proportional hazards regression. Candidate variants (red dots) met the following criteria: (i) unadjusted *P* < 0.05 in both replicates, (ii) consistent direction of effect across replicates, and (iii) FDR adjusted *P* < 0.01 when combining the two replicates. Noncandidate variants (gray dots) did not meet these criteria. (**B**) Comparison of absolute frequency differences (*y* axis) for candidate and noncandidate SNPs between pairwise samples with differing survival times. The “phenotypic difference level” refers to the difference in bin numbers of samples in the PCSS experiments. (**C**) Enrichment of candidate variants in functional genomic elements. SNPs were assigned to nine annotation groups, including coding (missense and synonymous), noncoding (UTR, intron, and intergenic), and regulatory regions (uORF, transcription factor binding sites, and miRNA target sites). The ES is the log_2_ scale of fold enrichment. (**D**) Enrichment of candidate SNPs in regulatory elements identified from modENCODE data, including transcription factor (TF) binding sites and regions near histone modifications. The top 20 most significant terms are shown. The ES is the log_2_ scale of fold enrichment. Asterisks indicate statistical significance using Fisher’s exact test (**, adjusted *P* < 0.01; ***, adjusted *P* < 0.001; n.s., adjusted *P* > 0.05).

To identify SNPs associated with starvation resistance, we applied a Cox proportional hazards regression model, transforming the counts of reference and alternative alleles to survival dataset, and identified SNPs that have higher risk of death under starvation (see Materials and Methods). In total, 57,433 SNPs (2L: 13,963; 2R: 13,416, 3L: 13,407; 3R: 12,083; 4: 11; X: 4,551; and Y: 2) were identified as candidates associated with starvation resistance (Fig. 2A).

To assess allele frequency shifts associated with phenotypic sorting, we analyzed the frequency of each genetic variant across seven phenotypic bins, which correspond to varying survival times from the PCSS experiments. For each variant, we calculated pairwise differences in allele frequencies between phenotypic bins (Fig. 2B). Given that the association between the reference and alternative alleles with starvation resistance is unknown, we focused on the absolute frequency differences. The “phenotypic difference level” refers to comparisons between two bins with varying survival times. For example, level 1 represents the comparison between neighboring bins, such as between the most sensitive group (bin 1) and the next one (bin 2), or between bin 4 and bin 5. Likewise, level 6 represents the comparison between the most sensitive (bin 1) and most resistant (bin 7) groups. The allele frequency differences of the same phenotypic difference level were pooled in the analysis. For candidate variants, absolute allele frequency differences increased steadily with greater phenotypic divergence, from a median of ~0.05 at level 1 to ~0.11 at level 6. In contrast, noncandidate variants showed relatively stable allele frequency differences (~0.04 to 0.06) across all levels. Statistical analysis confirmed that candidate variants exhibited significantly larger shifts than noncandidate variants (*P* < 0.001, Wilcoxon rank-sum test). These results suggest the polygenic architecture of starvation resistance (*29*–*35*), with many loci of small effect collectively contributing to the phenotype.

To assess whether genes associated with starvation resistance are adaptive in natural populations of *D. melanogaster*, we examined the overlap with positively selected genes (PSGs) identified in Chinese populations using three independent methods: Fay and Wu’s *H*, Ohana, and population branch excess (*39*). Of the genes containing SNPs detected in this study, 743 showed signatures of positive selection. The top 100 genes, ranked by their association strength with starvation resistance (adjusted *P*), were significantly enriched for PSGs (17 PSGs; fold enrichment, 3.93; *P* = 9.40 × 10^−6^, Fisher’s exact test). This enrichment remained significant for the top 200 and 500 genes (fold enrichment, >2; *P* < 0.001 for both). These results suggest that starvation resistance–associated variants are more likely to contribute to environmental adaptation, although some may be linked noncausally to the target variants through hitchhiking.

### Functional annotations of starvation-associated genetic variants

We calculated the enrichment score (ES; at the log_2_ scale) to quantify the overrepresentation or underrepresentation of candidate variants in each functional category relative to noncandidate variants (tables S4 and S5). We assessed the statistical significance of enrichment using Fisher’s exact test for each functional category and adjusted the *P* values with the Benjamini-Hochberg method. Compared to noncandidate variants, candidate variants were significantly underrepresented in introns (ES = −0.019, adjusted *P* = 8.50 × 10^−3^) and intergenic regions (ES = −0.096, adjusted *P* = 1.25 × 10^−22^) but overrepresented in synonymous (ES = 0.189, adjusted *P* = 1.37 × 10^−31^) and missense variants (ES = 0.238, adjusted *P* = 6.19 × 10^−15^).

We assigned candidate variants to specific genes if they fell within the genes’ coding regions, UTRs, or intronic regions and ranked these variants on the basis of their *P* values, with lower *P* values indicating higher significance. Upon examining the top 500 genes with the most significant candidate variants, we found that these genes are significantly enriched in biological processes such as “generation of neurons,” “animal organ morphogenesis,” “eye morphogenesis,” and “regulation of developmental process” (fig. S4). Gene ontology analysis of the top 500 genes with the most significant missense variants highlighted their involvement in processes such as “cyclic purine nucleotide metabolic process,” “positive regulation of protein localization to cell periphery,” “cAMP metabolic process,” and “peptide catabolic process” (fig. S5). Notably, *CG8060*, which encodes a protein that interacts with 4EBP [a key component of the insulin/insulin-like growth factor signaling (IIS) pathway] (*40*), had 19 candidate variants, including seven missense variants. As 4EBP plays an important role in regulating translation, growth, and stress responses (*41*), *CG8060* is likely involved in starvation response through these processes. In addition, *Lnk*, another gene in the IIS pathway, had three candidate variants, including two synonymous SNPs and one missense SNP (V58M).

To test for the enrichment of starvation-associated variants within specific regulatory categories, we analyzed the Model Organism Encyclopedia of DNA Elements (modENCODE) dataset (*42*). These candidate variants showed modest enrichment in transcription factor (TF) binding sites (ES = 0.034, adjusted *P* = 8.14 × 10^−6^) (Fig. 2C). To further investigate the regulatory impact of these variants, we analyzed their overlap with DNA regions bound by proteins or marked by histone modifications from the same dataset. Among 82 transcriptional regulation–related tracks, 25 were significantly enriched for these variants, while 7 had significantly fewer variants than expected [false discovery rate (FDR) < 0.05, Fisher’s exact test; Fig. 2D and table S5]. The most substantial enrichment was found in regions bound by *chinmo* (ES = 0.290, adjusted *P* = 8.14 × 10^−40^), a gene encoding a transcription factor involved in the response to caloric restriction (*43*). Binding sites for several other transcription factors, including Dll, Insv, Sens, and Fru, were also highly enriched. Regions associated with transcriptional activation, marked by RNA polymerase II binding sites and histone modifications such as H3K36me3, H3K4Me1, H3K4Me3, H3K27Ac, and H3K9Ac, were also enriched for the candidate variants. In contrast, fewer variants were detected in heterochromatin-associated regions, such as those marked by HP1 and H3K9Me3.

Notably, we also found an enrichment of candidate variants in the 5′UTR of genes in autosomes (ES = 0.105, adjusted *P* = 0.008) (table S4). In particular, we observed 87 candidate variants affecting the start codon of upstream open reading frames (uORFs) across 79 genes. These genes are functionally enriched in the pathways such as “pupal development” (e.g., *ash2*, *Mef2*, *shd*, and *ecd*), “tube development” (e.g., *Ptp61F*, *rols*, *TSG101*, and *sr*), “cell morphogenesis involved in neuron differentiation” (e.g., *dpr11*, *CG42327*, and *jim*), and “glycerolipid metabolism” (*CG8298*, *Gk2*, and *wun2*) (fig. S6). Given that uORFs can repress the translation initiation of the main coding sequence, and the start codon is a pivotal element of a uORF (*44*), these variants may influence the translational regulation of the main protein under starvation by disrupting or creating start codons of uORFs.

In summary, our functional enrichment analysis indicates that genetic variants associated with starvation resistance in *D. melanogaster* are significantly present in regions that alter protein sequences and in regulatory regions that control gene expression and translation. These findings suggest that both coding regions and regulatory sequences play crucial roles in the genetic architecture of starvation resistance.

### The V58M variant in *Lnk* increases starvation resistance with a sex-biased effect

**Fig. 3.**
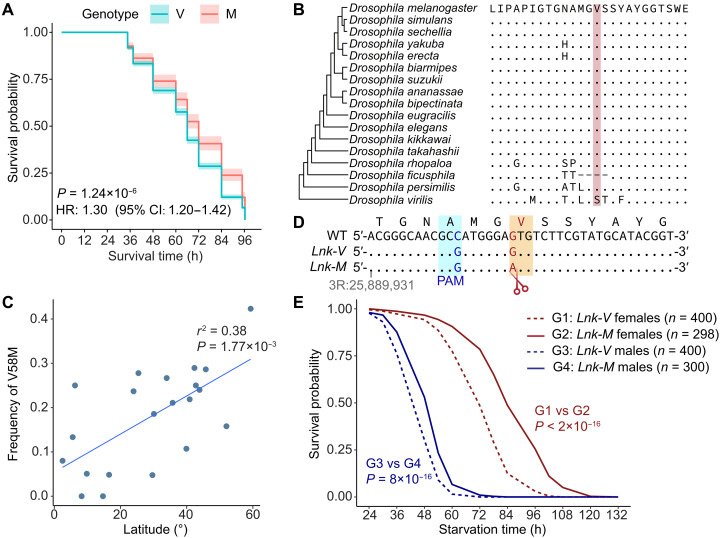
Impact of the V58M substitution in *Lnk* on starvation resistance. (**A**) Survival curves for the two alleles (V and M) at the 58th amino acid position of the Lnk protein. Survival probabilities for each allele are shown by solid lines, with 95% confidence intervals represented by the ribbon. The *P* values were calculated using the Cox proportional hazards regression model. The hazard ratio (HR) for the V allele and its 95% confidence interval (CI) are provided. (**B**) Multiple protein sequence alignment of the V58 site in Lnk across 17 *Drosophila* species. The alignment was taken from the UCSC Genome Browser. The conserved V58 site is highlighted in red (phyloP = 1.906, phastCons = 1). (**C**) Correlation between the frequencies of V58M and latitudes in 21 natural populations of *D. melanogaster* worldwide. The allele frequencies were obtained from a previous study (*39*). The *r*^2^ and *P* value of the linear regression using Pearson correlation are displayed. (**D**) Scheme depicting the generation of *Lnk-M* and *Lnk-V* genotypes by CRISPR-Cas9 technology. The mutations at the PAM site and at V58 are shown in blue and red, respectively. (**E**) Survival during starvation for *Lnk-V* and *Lnk-M* strains. Starvation resistance was measured in both males and females. The sample size (*n*) for each line is labeled. The *P* value for comparisons between *Lnk-M* (solid line) and *Lnk-V* (dashed line) was obtained using the log-rank test.

Lnk, an adaptor protein in the SH2B family, stabilizes the InR-chico interaction in the insulin-like receptor signaling pathway and plays key roles in cellular growth, as well as carbohydrate and triglyceride homeostasis (*45*, *46*). Our PCSS experiment identified a missense variant in *Lnk* (V58M) that is significantly associated with starvation resistance (adjusted *P* = 1.24 × 10^−6^) (Fig. 3A). Unexpectedly, despite the high conservation of the V58 residue across *D. melanogaster* and other *Drosophila* species (phyloP = 1.906, phastCons = 1; Fig. 3B), the V58M variant was polymorphic in natural populations of *D. melanogaster*, with frequencies ranging from 0 to 42% in the surveyed populations [table S6; data from ref. (*39*)]. The frequency of V58M was lower in sub-Saharan populations (mean: 12.0%; range: 0 to 27.8%) and higher in Europe, northern Africa, North America, and Oceania (mean: 25.4%; range: 15.8 to 42.7%). Among the Chinese strains, the frequency of V58M was low in the Qinghai-Tibet Plateau (4.8%) and high in Xinjiang (24%). Notably, the frequency of V58M showed a significant positive correlation with the latitude of the sample location (*r*^2^ = 0.38, *P* = 1.77 × 10^−3^; Fig. 3C), suggesting that this variant may contribute to environmental adaptation.

To validate the effect of the V58M variant, we used CRISPR-Cas9 to generate two allele-replacement strains: *Lnk-V* (V58 allele) and *Lnk-M* (M58 allele) (Fig. 3D). The genetic backgrounds of these strains were standardized over eight generations through crossing with the ISO-CS strain. Phenotypic assays on four *Lnk-V* and three *Lnk-M* homozygous mutant lines revealed that V58M significantly increased female starvation resistance. *Lnk-M* females had a mean survival time of 87.4 hours compared to 74.2 hours for *Lnk-V* [log_2_ fold change (log_2_FC) = 0.24, *P* < 2 × 10^−16^, log-rank test]. Males showed a similar pattern, with mean survival times of 51.7 hours for *Lnk-M* versus 47.1 hours for *Lnk-V* (log_2_FC = 0.13, *P* = 8 × 10^−12^, log-rank test) (Fig. 3E). An ANOVA (analysis of variance) revealed a significant interaction between sex and genotype (*P* = 5.05 × 10^−10^, table S7), suggesting a sex-biased effect of the V58M variant.

In conclusion, our findings show that the V58M variant in *Lnk* significantly increases starvation resistance in *D. melanogaster*. These results align with higher V58M frequencies in population cage samples with longer starvation survival times and natural populations at higher latitudes, where food shortages might be more severe. While the V58M variant increases starvation resistance in both sexes, it exhibits a sex-biased effect.

### Validating candidate genes in starvation resistance using RNAi

We conducted RNAi experiments on 131 candidate genes (135 RNAi strains) using a ubiquitous Gal4 driver (*Ubi-Gal4*) to assess the phenotypic effects on starvation resistance in adult *D. melanogaster* of both sexes. The candidate genes included 123 genes with at least 15 candidate variants or at least one missense candidate variant from the PCSS experiments, four genes with fewer than 15 candidate SNPs, and four additional genes identified in previous studies for their role in starvation response (table S8). Notably, 46 of these genes overlapped with a combined set of candidate genes from three previous studies [163 genes from ref. (*31*), 382 genes from ref. (*33*), and 292 genes from ref. (*34*)]. Given that our PCSS experiments show regulatory changes contributing to *Drosophila* starvation resistance, RNAi experiments can effectively evaluate the functional impact of some regulatory mutations. While RNAi may not fully replicate the effects of nonsynonymous mutations, it can mimic some effects by reducing protein activity if the gene is involved in starvation responses.

Knockdown of 25 candidate genes resulted in lethal or male-specific lethal phenotypes (Table 1 and table S8). Of these, 18 genes caused developmental failure during the embryo (eight genes), larval (one gene), or pupal (nine genes) stages. Knockdown of seven genes led to male-specific lethality and significantly reduced starvation resistance in female offspring (*P* < 0.001, log-rank test). Notably, among the male-specific lethal genes, knockdown of *Tep3* and *ds* also caused wing deformities in females. These findings suggest that the 25 candidate genes may play essential roles in *Drosophila* development. Seventeen genes showed consistent effects on starvation resistance in both sexes following RNAi knockdown without significant sex differences. Of these, 13 enhanced and 4 reduced starvation resistance (Table 1 and table S8). In contrast, 11 genes had no effect in either sex.

**Table 1.** RNAi phenotypes of candidate genes and sex-dependent effects on starvation resistance. Genes are grouped into three main categories: (i) lethal phenotypes, including embryonic, larval, pupal, and male-specific lethality; (ii) consistent effects in both sexes, further classified as enhancing or weakening starvation resistance; and (iii) sex-dependent effects (49 genes validated using qRT-PCR), including sex-specific (enhanced or weakened), sex-biased (enhanced or weakened), and sexually antagonistic effects. The number of genes and their names are provided for each category. Effect sizes, statistical significance, and qRT-PCR knockdown efficiencies are listed in table S9.

Category	No. of genes	Gene symbols
Lethal phenotypes		
Embryonic lethal	8	*Ssrp*, *Duox*, *nAChR*α*6*, *Dys*, *l(2)34Fc*, *Rep*, *krimp*, *Gprk2*
Larval lethal	1	*lilli*
Pupal lethal	9	*hth*, *pnt*, *sns*, *Atg4a*, *robo3*, *sls*, *Msp300*, *Apoltp*, *CG44837*
Male-specific lethal	7	*pum*, *fru*, *hdc*, *Dscam1*, *Tep3*, *ds*, *Ank2*
No phenotypic effect	11	*Sema-2a*, *dve*, *beat-IIIc*, *CG13185*, *CG5819*, *A2bp1*, *ppk11*, *dally*, *cpo*, *lov*, *Dop1R2*
Consistent effects in both sexes		
Increased resistance	13	*Ace*, *mud*, *ush*, *Apc*, *CadN*, *Rx*, *Tequila*, *eys*, *Daxx*, *CG15523*, *MTF-1*, *CG42232*, *Glut1*
Weakened resistance	4	*fz2*, *mtt*, *CG31690*, *Snoo*
Sex-dependent effects (49 genes validated using qRT-PCR)		
Female-specific (enhanced)	1	*CG15046*
Female-specific (weakened)	1	*mbt*
Female-biased (enhanced)	3	*CG4341*, *Pde6*, *dp*
Male-specific (enhanced)	5	*CG2712*, *htt*, *CG2975*, *Hipk*, *Eip93F*
Male-specific (weakened)	6	*CG2121*, *CG15117*, *Ncc69*, *PK2-R2*, *CG32264*, *Fhos*
Male-biased (enhanced)	8	*Gem3*, *Calx*, *drongo*, *CG8060*, *CG7362*, *CG3502*, *Pde11*, *St3*
Male-biased (weakened)	13	*fz*, *Sdc*, *CG18557*, *CG17568*, *CG17249*, *Hs6st*, *CG31145*, *fred*, *Ccn*, *Claspin*, *CG34347*, *psq*, *Pde8*
Sexually antagonistic (enhanced in females and weakened in males)	12	*ft*, *Ptp61F*, *GEFmeso*, *Lnk*, *Src64B*, *Gdh*, *bru-3*, *Ptp99A*, *fig*, *JHDM2*, *CG44153*, *Ten-m*

For the remaining genes that showed starvation resistance effects in at least one sex, we examined whether sex-biased RNAi knockdown efficiency might have confounded the observed sex-biased phenotypic effects. We used quantitative reverse transcription polymerase chain reaction (qRT-PCR) to quantify gene expression levels in 3- to 5-day-old adults of both sexes after knockdown using wild type as controls (Materials and Methods). qRT-PCR results were successfully obtained for 68 genes, of which 49 showed substantial knockdown in both sexes (table S9). The remaining 19 genes displayed unexpected knockdown patterns, including sex-specific knockdown, no notable change, or very low expression level in adults. Therefore, subsequent analyses focused on the 49 genes with robust down-regulation in both sexes after RNAi knockdown.

**Fig. 4.**
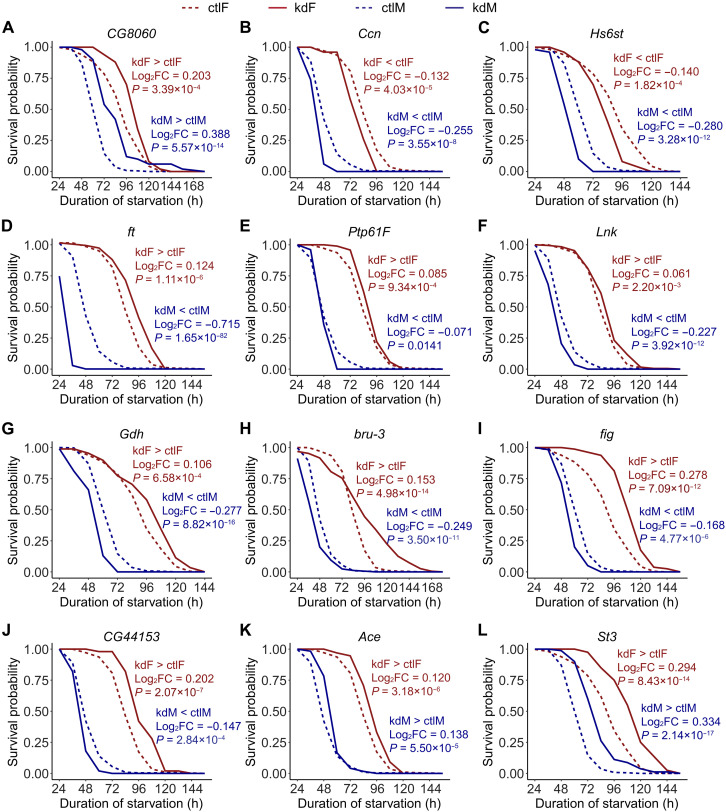
Survival curve under starvation for representative RNAi knockdowns versus matched controls. (**A**) Phenotypic consequences of *CG8060* RNAi knockdown, a top candidate gene for starvation resistance. *CG8060* encodes a protein interacting with 4EBP, a key regulator of the IIS pathway. (**B** and **C**) Examples from growth/Hippo–related genes (*Ccn* and *Hs6st*), part of a set of 13 genes functionally linked to growth control and Hippo signaling. (**D** to **F**) Sexually antagonistic genes in Hippo/IIS pathways: *ft*, *Ptp61F*, and *Lnk*. (**G**) *Gdh* (mitochondrial glutamate dehydrogenase) with sexual antagonism. (**H** to **J**) Additional antagonistic examples from diverse functions: *bru-3* (mRNA splicing/translation), *fig* (protein dephosphorylation), and *CG44153* (GPCR signaling), all enhancing female survival while reducing male survival. (**K** and **L**) Starvation survival of RNAi-targeting genes with pleiotropic effects, including *Ace* and *St3*. Knockdown of both genes showed increased survival under starvation, while down-regulation of *Ace* and *St3* decreased the insecticide resistance (*60*) and resistance to bacterial infection (*61*), respectively. For all panels, knockdown (solid lines) and control (dashed lines) survival curves are shown for females (kdF and ctlF) and males (kdM and ctlM) (*n* ≥ 50 per genotype and sex). The log_2_FC of mean survival time (knockdown versus control; positive values indicate increased survival) is indicated in each panel. *P* values are from log-rank tests comparing knockdowns to controls within sex.

Among these 49 genes, 38 knockdowns significantly affected starvation resistance in females and 47 in males. The disparity between male and female effects may reflect the male-focused design of our PCSS experiment. Overall, our RNAi experiments revealed substantial phenotypic changes associated with genes enriched for the most significant variants. For instance, the knockdown of *CG8060*, the gene with seven top candidate variants, extended mean starvation survival from 93.38 to 107.52 hours in females (log_2_FC = 0.20, *P* = 3.39 × 10^−4^, log-rank test) and from 66.75 to 87.36 hours in males (log_2_FC = 0.39, *P* = 5.57 × 10^−14^, log-rank test) (Fig. 4A).

To evaluate the potential off-target effects of RNAi, we examined *eys* using multiple RNAi strains (fig. S7). Silencing the *eys* gene with different RNAi strains consistently led to increased starvation resistance in the F1 offspring. This was observed across all three tested RNAi strains (THU4015, THU5876, and THU5878) in both male and female offspring (all six tests showed a statistically significant increase with adjusted *P* < 0.05 using the log-rank test). Although the efficiency of gene silencing may vary between different RNAi strains, the reproducible phenotype across independent RNAi constructs strongly suggests that the observed effect is specific to *eys* and not due to off-target artifacts.

### Widespread sex-specific, sex-biased, and sexually antagonistic effects in starvation resistance genes

Among the 49 genes showing robust down-regulation in both sexes after RNAi knockdown, we observed diverse effects on starvation resistance, including sex-specific, sex-biased, and sexually antagonistic phenotypes (Table 1 and table S9). Two genes exhibited female-specific effects: *CG15046* increased and *mbt* decreased starvation resistance. Eleven genes showed male-specific effects, with five enhancing and six reducing resistance. Sex-biased effects were more prevalent in males: 21 genes exhibited significantly stronger effects in males (8 with greater enhancement and 13 with greater reduction), whereas only three genes showed stronger enhancement in females. Notably, 12 genes displayed sexually antagonistic effects, all of which increased starvation resistance in females but decreased it in males. Representative examples include *ft*, *Ptp61F*, and *Lnk*, which are involved in the Hippo and IIS pathways and may be subject to opposing selective pressures between the sexes.

Gene ontology analysis of these 49 genes revealed enrichment in several biological processes, including “regulation of intracellular signal transduction,” “regulation of cAMP/PKA signal transduction,” and “regulation of growth” (fig. S8). Thirteen of these genes (*mbt*, *ft*, *Sdc*, *Hipk*, *Ptp61F*, *Hs6st*, *Lnk*, *fz*, *Src64B*, *fred*, *Ccn*, *CG2121*, and *Ten-m*) are functionally related to the regulation of growth and the Hippo signaling pathway (Fig. 4, B and C, and fig. S9), a crucial regulator of cell proliferation and organ size (*47*). Intriguingly, five of these genes (*ft*, *Ptp61F*, *Ten-m*, *Lnk*, and *Src64B*) displayed sexually antagonistic effects, whereby RNAi knockdown enhanced starvation resistance in females but weakened it in males. For example, *ft*, which encodes a transmembrane protein that limits cell growth, harbored 19 candidate variants associated with starvation resistance. The knockdown of *ft* increased mean female starvation survival by 7.99 hours (log_2_FC = 0.12, *P* = 1.11 × 10^−6^, log-rank test) but decreased mean male starvation survival by 21.26 hours (log_2_FC = −0.71, *P* = 1.65 × 10^−82^, log-rank test) (Fig. 4D). *Ptp61F*, encoding a tyrosine phosphatase that regulates the Hippo and IIS pathways, harbored 184 candidate variants associated with starvation resistance. Knocking down *Ptp61F* increased mean female starvation survival by 5.36 hours (log_2_FC = 0.08, *P* = 0.0009, log-rank test) but decreased mean male starvation survival by 2.61 hours (log_2_FC = −0.07, *P* = 0.014, log-rank test) (Fig. 4E). Similarly, knocking down *Lnk*, another gene in the IIS pathway, increased mean female starvation survival by 3.85 hours (log_2_FC = 0.06, *P* = 0.002, log-rank test) but decreased mean male starvation survival by 7.91 hours (log_2_FC = −0.23, *P* = 3.92 × 10^−12^, log-rank test) (Fig. 4F). Given that the Hippo and IIS pathways are functionally connected in growth control and metabolic regulation, these findings suggest that their components—individually and through their regulatory interactions—are subject to opposing selective pressures in males and females, potentially contributing to the maintenance of genetic variation in traits related to stress resistance.

We also observed sexual conflict in genes with other functions. For instance, *Gdh*, which encodes a glutamate dehydrogenase in mitochondria and is associated with starvation resistance (*33*), had four candidate variants. The knockdown of *Gdh* increased mean female starvation survival by 7.12 hours (log_2_FC = 0.11, *P* = 6.58 × 10^−4^, log-rank test) but decreased mean male starvation survival by 11.6 hours (log_2_FC = −0.28, *P* = 8.82 × 10^−16^, log-rank test) (Fig. 4G). Other genes with sexual antagonistic effects on starvation resistance have functions related to mRNA splicing and translation (*bru-3*), protein dephosphorylation (*Ptp99A* and *fig*), histone demethylation (*JHDM2*), or G protein (heterotrimeric guanine nucleotide–binding protein)–coupled receptor signaling (*CG44153*). The knockdown of these genes all resulted in enhanced starvation resistance in females and weakened starvation resistance in males (Fig. 4, H to J, and fig. S9).

In summary, among the 77 candidate genes without lethal effects, 66 (85.7%) showed evidence of influencing starvation resistance, including 49 with sex-dependent effects validated by qRT-PCR and 17 with consistent effects in both sexes. We did not perform qRT-PCR validation for the 17 consistently acting genes, as their robust phenotypes strongly suggest effective knockdown. For the 11 genes with no detectable phenotypic effects, qRT-PCR was also not performed; some of these may have lacked observable effects because of inefficient knockdown, suggesting that our estimate of functionally relevant genes may be conservative. Notably, 49 of the 66 (74.2%) starvation-associated genes exhibited sex-biased effects, including 5 with female-specific or female-biased responses, 32 with male-specific or male-biased responses, and 12 with sexually antagonistic effects. Notably, all 12 antagonistic cases involved increased starvation resistance in females and decreased resistance in males following RNAi knockdown, suggesting that the wild-type (endogenous) function of these genes may preferentially benefit males but impose a cost on females under starvation conditions.

### Pleiotropic effects and trade-offs in starvation resistance and stress response genes

We found evidence of pleiotropic effects in two starvation resistance genes. The *Ace* gene, with 17 candidate variants, is associated with starvation and insecticide resistance. In addition, the *St3* gene, with nine variants, plays a role in bacterial defense. Although the high expression of these genes may aid in insecticide resistance or bacterial defense, their knockdown increased starvation survival in both sexes. The knockdown of *Ace* increased mean starvation survival from 88.87 to 96.55 hours in females (log_2_FC = 0.12, *P* = 3.18 × 10^−6^, log-rank test) and from 54.43 to 59.9 hours in males (log_2_FC = 0.14, *P* = 5.50 × 10^−5^, log-rank test) (Fig. 4K), with no significant sex difference in the magnitude of the effect. *St3* knockdown increased mean starvation survival from 93.38 to 114.45 hours in females (log_2_FC = 0.29, *P* = 8.43 × 10^−14^, log-rank test) and from 66.75 to 84.15 hours in males (log_2_FC = 0.33, *P* = 2.14 × 10^−17^, log-rank test) (Fig. 4L). Cox proportional hazards regression revealed a modest but statistically significant stronger effect of RNAi knockdown in males (*P* < 0.01), classifying *St3* as a male-biased gene influencing starvation resistance. These findings highlight gene pleiotropy underlying starvation resistance genetic architecture and reveal evolutionary compromises in responding to diverse environmental stressors.

### Sex-biased expression and knockdown efficiency cannot account for sex-dependent starvation resistance

**Fig. 5.**
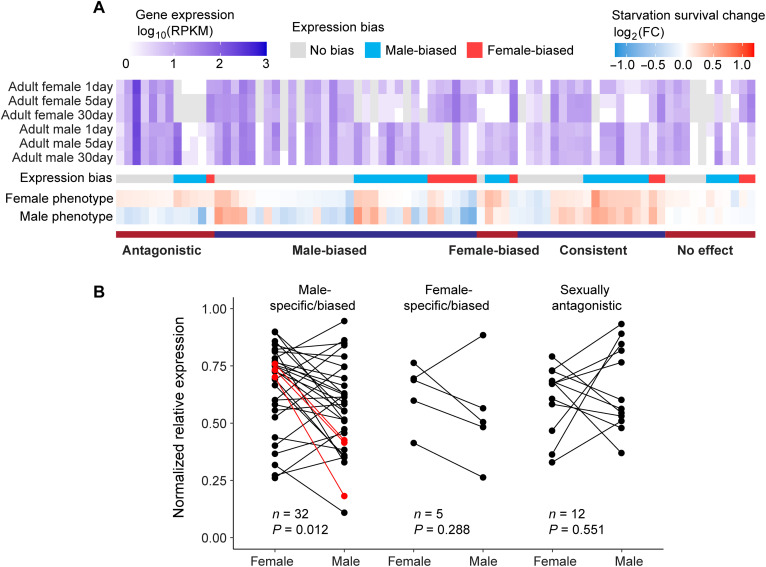
Pattern of sex-biased gene expression and RNAi knockdown efficiency. (**A**) Testing the relationship between gene expression bias and RNAi knockdown effects on starvation survival in females and males. Gene expression levels (RPKM, log_10_ scale) of the 77 genes in female and male adult whole bodies at 1, 5, and 30 days posteclosion are shown in the upper panel. Genes are categorized on the basis of the female/male expression ratio into three groups: male-biased (blue), female-biased (red), and no bias (gray), as shown in the middle bar. In the lower panel, the phenotypic effect of each RNAi knockdown is displayed. The mean survival time under starvation for each knockdown is divided by that of its control and shown on a logarithmic scale (log_2_FC). The bar below indicates the category of each candidate gene. (**B**) RNAi knockdown efficiency between sexes, shown as the normalized expression level for the three categories of sex-dependent genes (*n* = 49). Categories include male-specific/biased (*n* = 32), female-specific/biased (*n* = 5), and sexually antagonistic (*n* = 12). Paired *t* tests were used to detect sex biases in knockdown efficiency. In the male-specific/biased category, red dots represent the three genes that exhibited significantly lower expression in males after knockdown (adjusted *P* < 0.05). After excluding these three genes, the male-biased knockdown efficiency was no longer significant (*P* = 0.065, paired *t* test).

Sex-biased gene expression has been suggested as a mechanism to resolve sexual conflict (*48*–*53*). To examine whether the sex-specific, sex-biased, or antagonistic effects observed in starvation resistance are due to sex-biased gene expression, we analyzed baseline transcriptomic profiles from adult *Drosophila* using modENCODE data (*42*). Genes with female-to-male RPKM (reads per kilobase per million mapped reads) ratios >2 were classified as female-biased and those <0.5 as male-biased, as previously described (*54*). Among the 77 RNAi-targeted genes, 39 (50.6%) showed sex-biased expression—12 female-biased and 27 male-biased—while 38 showed no bias. Categorizing these genes into five phenotypic classes (antagonistic, consistent, female-biased, male-biased, and no effect; Fig. 5A) revealed no significant association between gene expression bias and phenotypic outcome (*P* > 0.05, Fisher-Freeman-Halton test; table S10). These data suggest that sex-biased gene expression does not predict phenotypic outcomes under expression disturbance.

To test whether RNAi knockdown efficiency differed between sexes and could account for phenotypic effects, we quantified postknockdown gene expression via qRT-PCR for the 49 genes showing robust knockdown (Fig. 5B). Paired *t* tests within phenotypic categories revealed no sex bias in knockdown efficiency among antagonistic (*n* = 12, *P* = 0.55) or female-biased (*n* = 5, *P* = 0.29) genes. In contrast, genes with male-biased phenotypic effects (*n* = 32) showed significantly stronger knockdown in males (*P* = 0.012; Fig. 5B). Gene-level *t* tests, followed by FDR correction (FDR < 0.05), identified three male-biased genes—*Sdc* (TH02042.N, adjusted *P* = 0.033), *CG8060* (TH03530.N, adjusted *P* = 0.013), and *CG17249* (TH05154.N, adjusted *P* = 0.013)—with significantly stronger knockdown in males. After excluding these three genes, the male-biased group no longer exhibited significant knockdown asymmetry (*P* = 0.065). Collectively, these results indicate that while sex differences in RNAi knockdown efficiency exist, they are unlikely to drive the observed sex-dependent phenotypic effects. In particular, antagonistic and female-biased effects are not explained by knockdown efficiency, and only a small subset of male-biased genes (3 of 32) showed differences large enough to influence the phenotype.

## DISCUSSION

This study identifies a large number of genetic variants associated with starvation resistance in *D. melanogaster* using the PCSS approach. Many of these variants are located in regulatory regions or alter protein sequences. RNAi knockdown experiments confirmed the relevance of these variants, with ~85.7% (66 of 77) of the candidate genes showing significant effects on starvation resistance. A total of 74.2% (49 of 66) of the genes exhibiting verified starvation effects demonstrated sex-specific, sex-biased, or sexually antagonistic responses. Twelve genes showed sexually antagonistic effects, enhancing starvation resistance in females while decreasing it in males. This suggests that the wild-type (endogenous) function of these genes may preferentially benefit males but impose a cost on females under starvation conditions.

**Fig. 6.**
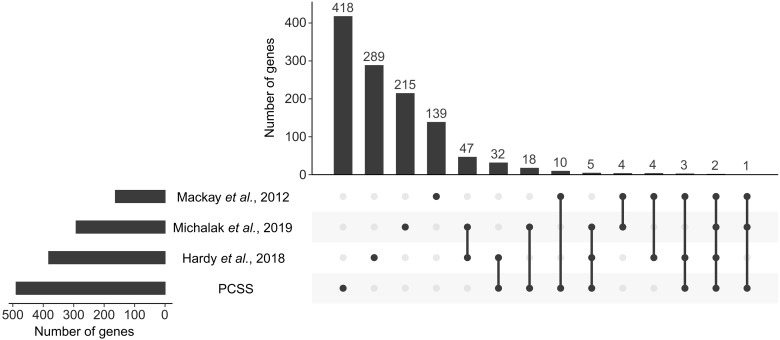
Overlap of candidate genes associated with starvation resistance across studies. Three previous studies identified candidate genes related to starvation resistance in *Drosophila melanogaster*: Mackay *et al.* (*31*) identified 163 genes, Hardy *et al.* (*33*) identified 382 genes, and Michalak *et al.* (*34*) identified 292 genes. Using the top 1000 SNPs with the strongest association signals, our PCSS approach identified 489 candidate genes. This diagram illustrates the overlap of candidate genes identified across these four studies.

Our findings support a polygenic architecture of multiple loci with small to moderate effects on starvation resistance in natural populations of *D. melanogaster*. The candidate SNPs associated with starvation resistance in our PCSS experiments were significantly enriched in PSGs at the population level. This suggests that variants associated with starvation resistance are more likely to contribute to environmental adaptation. However, some variants may be linked noncausally to target variants via hitchhiking. In addition, of the 489 candidate genes identified in our PCSS analysis, 14.5% (71 of 489) overlapped with previously reported genes (Fig. 6) (*29*–*35*), indicating a complex and heterogeneous genetic architecture for starvation resistance. This complexity arises from multiple selection trajectories influenced by genetic background, evolutionary history, and selection pressures.

Genes related to starvation resistance involve processes such as the IIS and Hippo pathways, which regulate metabolism, cell growth, organ size, and tissue homeostasis (*29*, *30*, *33*, *55*). Previous studies have shown significant associations between starvation resistance and key IIS pathway genes like *InR* and *foxo* (*33*, *34*, *56*–*59*). Consistent with these findings, our PCSS experiments detected strong associations in *InR* (5 candidate variants) and *foxo* (17 candidate variants). We also identified significant signals in other IIS genes, including *Ptp61F*, *Lnk*, *chico*, and *ush*. Our RNAi knockdown experiments revealed that five genes (*ft*, *Ptp61F*, *Ten-m*, *Lnk*, and *Src64B*) in the growth regulation and Hippo pathways exhibited sexually antagonistic effects, enhancing starvation resistance in females but weakening it in males. These findings suggest that sexual antagonism in these genes may be tied to nutrient storage, metabolic rate, body size, and lifespan, further connecting these processes to starvation resistance. However, because complex traits like starvation resistance involve many loci and limited overlap of starvation resistance genes was found across studies (Fig. 6), it is clear that the genetic background can strongly influence trait architecture. Determining how robust these sex-biased and sexually antagonistic effects are across diverse genetic contexts will require further investigation.

The evolution of starvation resistance often coincides with changes in other phenotypes, indicating overlapping genetic architectures and pleiotropic effects. Trade-offs between fitness-related traits are common, with alleles beneficial for one trait potentially detrimental to another. Our results support this scenario, with evidence from RNAi results for insecticide resistance (*Ace*) and defense response genes (*St3*). Elevated expression of *Ace* (encoding acetylcholinesterase) is associated with increased insecticide resistance in many insect species (*60*). In our study, the knockdown of *Ace* significantly increased starvation survival in both sexes, suggesting a potential trade-off between starvation resistance and insecticide resistance. Similarly, *St3*, involved in resistance to *Listeria monocytogenes* infection (*61*), also showed a significant increase in starvation survival upon knockdown in both sexes, indicating a possible trade-off between its immune function and starvation resistance. More broadly, such trade-offs in fitness-related traits may commonly exist between sexes, different environments, and developmental stages (*62*, *63*), resulting in complex relationships between genotypes and phenotypes.

Sexual antagonism has important evolutionary implications because it imposes opposing selection pressures on males and females for the same genetic variants. Such a conflict can slow or constrain adaptation by driving phenotypes toward a compromise that is suboptimal for both sexes, yet it can also maintain alleles at intermediate frequencies via balancing selection, preserving standing genetic variation that may be rapidly mobilized under changing environmental conditions. While we found that starvation-associated genes are generally enriched with PSGs at the population level, the role of sexually antagonistic loci in shaping adaptation trajectories remains unclear. Notably, our *Lnk* case study provides insights into this issue. While the RNAi knockdown of *Lnk* exhibits sexually antagonistic effects, the nonsynonymous V58M mutation shows similar but biased effects on starvation resistance. This suggests that different types of mutations within the same gene can lead to distinct phenotypic outcomes. In natural populations, combinatorial mutations may collectively contribute to adaptive responses. In addition, although the V58M mutation exhibits a frequency cline with latitude—suggesting natural selection—its maintenance at low-to-intermediate frequencies (0 to 40%) indicates potential sexual antagonisms or even pleiotropic effects beyond starvation adaptation.

The insights from this study extend beyond *Drosophila* and may have implications for understanding human diseases. In humans, sexually antagonistic alleles show differences in allele frequencies between sexes (*64*), sex-specific genetic architectures of complex traits and diseases (*65*, *66*), and sex-opposite effects for genetic variants (*67*). The presence of such antagonistic effects in key regulatory pathways like IIS and Hippo suggests a potential link to metabolic and growth-related disorders, as well as diseases influenced by nutrient storage and lifespan. Similar cases of sexual conflict have been reported in other species, such as Atlantic salmon (*68*) and plants (*69*), highlighting the broad relevance of these findings.

In summary, our findings highlight the complexity of fitness-related traits like starvation resistance, governed by polygenic traits with varying effects across sexes. These insights contribute valuable knowledge to evolutionary biology and have implications for breeding programs, conservation genetics, and human health.

## MATERIALS AND METHODS

### Population cage sorting and sequencing

We developed a cost-efficient method called PCSS to identify variants associated with starvation resistance. The advantages of the PCSS method lie in its utilization of population cages, notably enhancing throughput (allowing testing of up to 200 strains per trial) and simulating the natural competitive state of fruit flies in the wild. In addition, the method ensures uniform experimental conditions, reducing batch effects. In the PCSS, 200 isofemale strains were used to represent the natural populations of *D. melanogaster* in China (Fig. 1A and table S1). These strains were kept in the incubator under uniform conditions (25°C, 50% humidity, 12-hour light-dark cycle) for one generation. Parental flies from each of these strains were allowed to lay eggs in a vial with fresh cornmeal food and removed from the vial after 24 hours. In the offspring, 10 male adults at the age of 3 to 5 days after eclosion were collected from each strain and pooled into a population cage with cornmeal food to acclimate for 1 day. The medium was replaced by 1.5% agar to start the starvation stress. Dead individuals were collected from the cage and quickly frozen in liquid nitrogen with a time interval of 6 or 12 hours. The experiments were performed for two replicates. Individuals were grouped on the basis of the survival time into seven samples (A1 to A7 and B1 to B7). Seven representative samples with ~200 individuals from each replicate were sequenced to a depth of ~200×. We also sequenced two background samples (C1 and C2) to a depth of ~400×, each containing five males of each strain (around 1000 males in total). The survival time and the number of individuals for each sample are shown in Fig. 1B.

### Variant calling and filtration

Using BWA-MEM version 0.7.15 (*70*), we competitively mapped reads from each sample to the reference genomes of *D. melanogaster* (Flybase dmel_r6.24) and *Drosophila simulans* (dsim_r2.02) with the default parameters. Optical and PCR duplicates were marked by Picard version 2.20.5 (http://broadinstitute.github.io/picard). Variants were jointly called by freebayes version 1.3.5 (*71*) in a frequency-based mode (-C 1 --pooled-continuous). The parameters used include a minimum mapping quality of 20, a minimum base quality of 20, a haplotype length of 0 (-m 20 -q 20 --haplotype-length 0), and a minimum frequency of 0.01 (-F 0.01). We removed variants with a quality below 20. Multiallelic variants were split by bcftools version 1.9 norm (*72*). The multiple-nucleotide polymorphism was decomposed using vcfallelicprimitives in vcflib (*73*). Repeat regions and low-complexity DNA sequences were masked using the RepeatMasker track in the UCSC Genome Browser (http://genome.ucsc.edu/).

In the high-depth pooled sequencing, rare variants can be caused by sequencing errors. According to previous research, raising the threshold of supportive read counts in variant calling can effectively reduce the false positive rate (*38*). To obtain a high-quality variant dataset, we focused on background samples C1 and C2 and filtered the variants with the following criteria: (i) The minor allele counts in total should be more than five for autosome variants and more than two for X chromosome variants, and (ii) the loci with a depth at the extreme 1% (1035 for autosomes and 531 for the X chromosome) could be vulnerable to mismapping and are excluded from further analysis. In addition, the loci with a sequencing depth lower than 50 in each sample were also removed for they may not provide enough information. Furthermore, indels and variants within 3 bp of indels were removed from the analysis. In total, 1,409,919 high-quality variants were detected in background samples (C1 and C2), and more than 98.4% of variants were detected in each of the other samples. To perform the regression analysis, we additionally required that variants should be detected in at least one sample for both replicates. After the filtration, 1,285,620 variants (2L: 319,115; 2R: 250,466; 3L: 282,560; 3R: 306,850; 4: 552; X: 125,926; Y: 151) were retained and used for subsequent analysis. After variant calling and filtration, all variants were annotated with SnpEff (*74*) on the basis of the BDGP6.86 library. For each genetic variant, if multiple annotations were present, we retained the one with the most severe predicted effect, following this priority: missense mutations > synonymous mutations > UTR variants > intronic variants > intergenic variants.

### Candidate variant detection

To identify genetic variants associated with starvation resistance, we applied a Cox proportional hazards regression model (*75*) implemented in the R package survival (*76*). For each SNP, the read counts of the reference and alternative alleles were transformed into survival data. Specifically, if the reference allele count was *m* and the alternative allele count was *n* in sample A1, then (*m* + *n*) rows of survival data were generated: *m* rows labeled “REF” and *n* rows labeled “ALT” in the genotype (GT) column, with all rows assigned the mean starvation survival time of sample A1 in the “time” column. This process was repeated for all samples.

The Cox model (coxph) was fitted either to each replicate separately or to both replicates combined using the formula time ~ GT + replicate. *P* values were adjusted for multiple testing using the Benjamini-Hochberg method. Variants were considered associated with starvation resistance if they met all of the following criteria: (i) unadjusted *P* < 0.05 in both replicates; (ii) consistent direction of effect across replicates; and (iii) adjusted *P* < 0.01 in the combined model. Using these criteria, we identified 57,433 candidate variants putatively associated with starvation resistance.

### Functional enrichment analysis

We annotated missense, synonymous, 5′UTR, 3′UTR, intronic, and intergenic variants using SnpEff (*74*). uORF variants were annotated by UTRannotator (*77*) implemented in VEP version 108.2 (*78*). Only variants annotated as “uAUG gained” or “uAUG lost” were considered as uORF variants. miRNA (microRNA) target sites were derived from TargetScan (release 7.2) (*79*). Regulatory regions such as transcription factor binding sites and genomic regulatory regions marked by histone modification and other epigenetic factors were obtained from modENCODE datasets (*42*). For factors with more than two modENCODE terms, we require that sites associated with this factor be supported in at least two terms. The enrichment analyses were conducted by comparing the proportion of certain types of sites in the candidate set to their proportion in the background set, and the significance was assessed using Fisher’s exact test. The adjusted *P* values were calculated using the Benjamini-Hochberg method.

Gene ontology analysis was performed using Metascape (*80*), focusing on biological processes and Kyoto Encyclopedia of Genes and Genomes pathways. Genes harboring SNPs in the PCSS data were included in the background gene set. Enrichment criteria included (i) a minimum of three overlapping genes, (ii) *P* value <0.01, and (iii) a minimum fold enrichment of 1.5.

### Construction of *Lnk* mutant strains using CRISPR-Cas9

We carried out CRISPR-Cas9–mediated homology-directed repair (*81*) to introduce the V58M (GTG to ATG) point mutation in *Lnk*. We chose the optimal single guide RNA (sgRNA) with high specificity and low off-target effects with Benchling’s CRISPR design tool (www.benchling.com/crispr/). Single-stranded complementary DNAs were synthesized and annealed to obtain double-stranded DNA, and the double-stranded DNA was ligated into the Bbs I–digested pU6B vector. The repair donor plasmids with about 2 kb flanking the target site into the pMD19-T vector were constructed following the methods described in the previous study (*81*). We also introduced a synonymous mutation (GCC to GCG) into the PAM sequence to prevent Cas9 from continuing to target the repaired DNA (Fig. 3D). Two kinds of donor plasmids were constructed. The first type contained mutations in both the target site and PAM site (*Lnk-M*), while the second type had mutations only in the PAM site (*Lnk-V*), serving as a control. The repair donor plasmid and pU6B-sgRNA plasmid were co-injected into Cas9 transgenic fly (TH00788.N) embryos, and the injected embryos were kept at 25°C and 60% humidity until adulthood (G0) for screening (*82*). The plasmid backbones, toolkit strains, and microinjection service are provided by the Tsinghua Fly Center (THFC). The screening of mutant flies was conducted according to the protocol described in a previous study (*82*). The G0 adults were crossed with TB00077 (*y sc v*), and the F1 offspring were crossed with TB00139 (*y sc v;; Dr,e/TM3,Sb*) because the target is on 3R. After spawning, F1 individuals were genotyped by PCR amplification of target loci and Sanger sequencing to select individuals with mutations of interest. Then, F2 males with the *yellow^−^* and *TM3* phenotype were crossed with TB00139, and F2 males were screened by genotyping. F3 offspring with the same mutation were crossed to generate the homozygote mutant strain. Then, the flies were backcrossed eight times to Canton-S flies to achieve an isogenic background. The sequence of sgRNA and other primers used for plasmid construction and genotyping are listed in table S11.

### *Drosophila* stocks used in RNAi experiments

We obtained 135 transgenic RNAi lines from the Tsinghua Fly Center (THFC) targeting 131 candidate genes (table S8). Each line carries an inducible short hairpin RNA under the control of a UAS promoter. To induce knockdown, UAS-RNAi lines were crossed to a ubiquitously expressed Gal4 driver (*Ubi-Gal4*, TB00152), and F1 offspring were assayed for starvation resistance. For lines carrying a balancer chromosome, only F1 flies lacking the balancer were used. To control for genetic background effects in RNAi experiments, we used background-matched control crosses for each RNAi line. Specifically, TB00072 (*y v; attP2, y^+^*) was used as the control for RNAi lines with constructs inserted at the *attP2* site, and TB00073 (*y v; attP40, y^+^*) served as the control for lines inserted at the *attP40* site. Each RNAi line was crossed to the *Ubi-Gal4* driver line, and for each gene, the corresponding control cross was generated by mating the appropriate background line (TB00072 or TB00073) to the same *Ubi-Gal4* driver. F1 progenies from these background-control crosses were used as negative controls for RNAi knockdown, ensuring that any phenotypic differences were attributable to gene-specific knockdown rather than insertion site or genetic background.

### Starvation resistance assays

Fruit flies were fed with the starvation diet and assessed for survival time. The starvation medium consisted of 1.5% agar and 5 ml of distilled water in each culture vial. For *Lnk-M* and *Lnk-V* strains, parental flies (30 pairs) were allowed to lay eggs in the bottle with the standard cornmeal medium and were removed from the bottle after 24 hours to control the density. For RNAi strains, five *Ubi-Gal4* virgin females and three UAS-RNAi males per vial were crossed and left in vials for 24 hours. Nonvirgin flies at the age of 3 to 5 days after eclosion were collected from each strain and separated by sex before placement on the starvation medium. When recording starvation survival time, 10 flies were placed in each vial, and the number of survivals was counted every 12 hours. No less than five replicate vials were assayed for each sex and genotype. These procedures were all conducted under conditions of 25°C, 50% humidity, and a 12-hour light-dark cycle. The survival curves of different strains were compared by the log-rank test. In total, we measured the starvation resistance for about 16,800 adults.

### Sex-dependent effects on starvation resistance

For each gene, we applied a Cox proportional hazards regression model (*75*) implemented in the R package survival (*76*): cox_model <- coxph(Surv(time, death_state) ~ genotype * gender, data = data). The model included the interaction term between genotype (knockdown versus control) and sex to test for sex-dependent effects. Because females generally exhibited substantially longer survival times than males, absolute changes in survival time were not directly comparable between sexes. To account for this baseline difference, we evaluated sex effects using the fold change in survival time relative to the sex-specific control rather than absolute differences in hours. Sex-biased effects were defined using two criteria. First, if a gene showed a statistically significant phenotype (*P* < 0.05, log-rank test) in only one sex, it was classified as sex-specific regardless of the interaction term. Second, for genes with consistent directional effects in both sexes (e.g., both enhanced or both reduced survival), we used the significance of the genotype-by-sex interaction (*P* < 0.05) to infer sex bias. Genes that exhibited opposing phenotypic effects in males and females (e.g., enhanced in females and weakened in males) were classified as showing sexual antagonism. To correct for multiple testing, *P* values were adjusted using the Benjamini-Hochberg method. For the 49 genes with qRT-PCR–validated knockdown, all FDR values were below 0.038.

### Validation of RNAi knockdown efficiency across sexes using qRT-PCR

To evaluate RNAi knockdown efficiency in both sexes, gene expression was quantified by qRT-PCR in F1 male and female adults from each RNAi cross. For each line, three biological replicates per sex were collected, with each replicate consisting of 20 adults aged 3 to 5 days. Total RNA was extracted using TRIzol reagent (Invitrogen), treated with deoxyribonuclease to remove genomic DNA contamination, and reverse transcribed using the PrimeScript II kit (Takara). qRT-PCR was performed with PowerUp SYBR Green Master Mix (Applied Biosystems) using *RpL32* as the endogenous control. Each biological replicate was run in duplicate (technical replicates). Knockdown efficiency was calculated using the comparative *C*_T_ method. Primer sequences are provided in table S12. To ensure consistency with the RNAi phenotyping experiments, the control lines used in the qRT-PCR assays matched the genetic background of each RNAi line: TB00072 (*attP2*) or TB00073 (*attP40*). These background-matched controls were crossed to the same *Ubi-Gal4* driver as the experimental RNAi lines, and F1 progenies were used as negative controls. The statistical significance of gene expression differences between knockdown and control groups was evaluated using two-sided Student’s *t* tests.

### Statistical analyses

All statistical analyses were performed using R (www.r-project.org).

## Supplementary Materials

### The PDF file includes:

Figs. S1 to S9 Legends for tables S1, S5, S8, S9, and S12 References
