# Supplementary material for: Pervasive sex-dependent effects in the genetic architecture of starvation resistance in Drosophila melanogaster
Source: Sci Adv. 2025 Sep 26;11(39):eadt3560. doi: 10.1126/sciadv.adt3560 (PMC12466915; doi:10.1126/sciadv.adt3560)
Supplement: Supplementary file 1 — Figs. S1 to S9 Legends for tables S1, S5, S8, S9, and S12 References [file sciadv.adt3560_sm.pdf]

Science Advances
AAAS

Supplementary Materials for

Pervasive sex-dependent effects in the genetic architecture of starvation resistance in *Drosophila melanogaster*

Junhao Chen *et al.*

Corresponding author: Jian Lu, luj@pku.edu.cn

Sci. Adv. **11**, eadt3560 (2025)
DOI: 10.1126/sciadv.adt3560

The PDF file includes:

Figs. S1 to S9
Legends for tables S1, S5, S8, S9, and S12
References

Other Supplementary Material for this manuscript includes the following:

Tables S1, S5, S8, S9, and S12

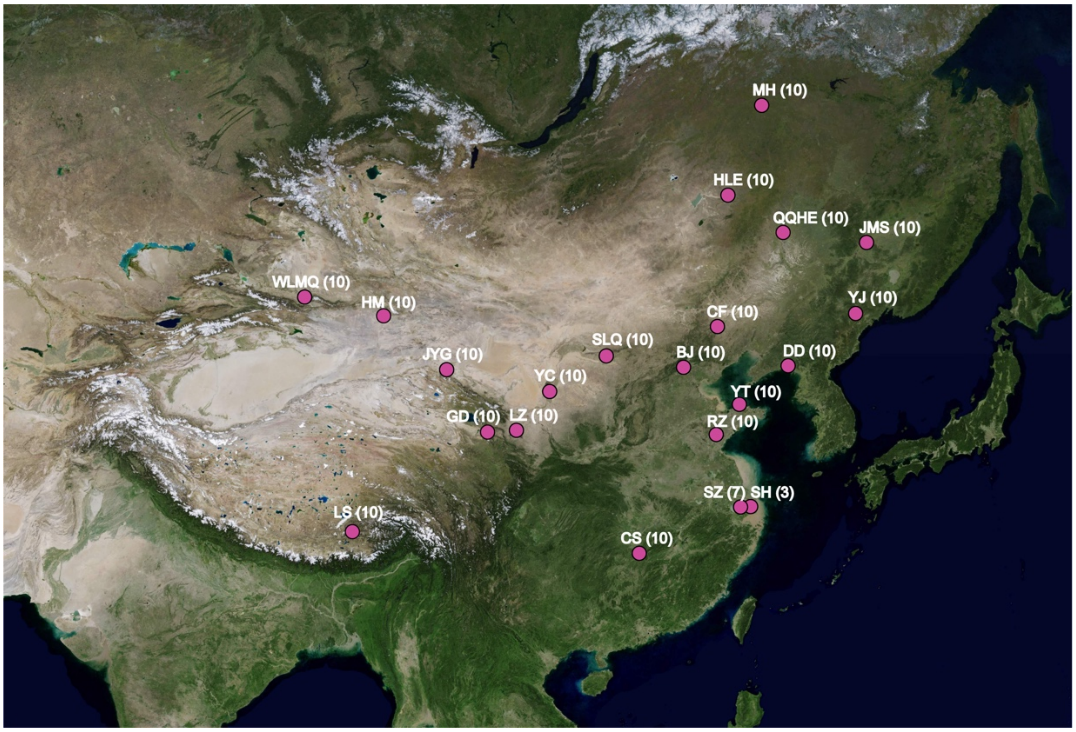

Fig. S1. Geographic locations of 200 strains collected from 21 cities in China. The purple dots denote sampling locations, with the number in parentheses indicating the sample size of strains used at each location. The detailed information of sampling locations and strains are shown in table S1.

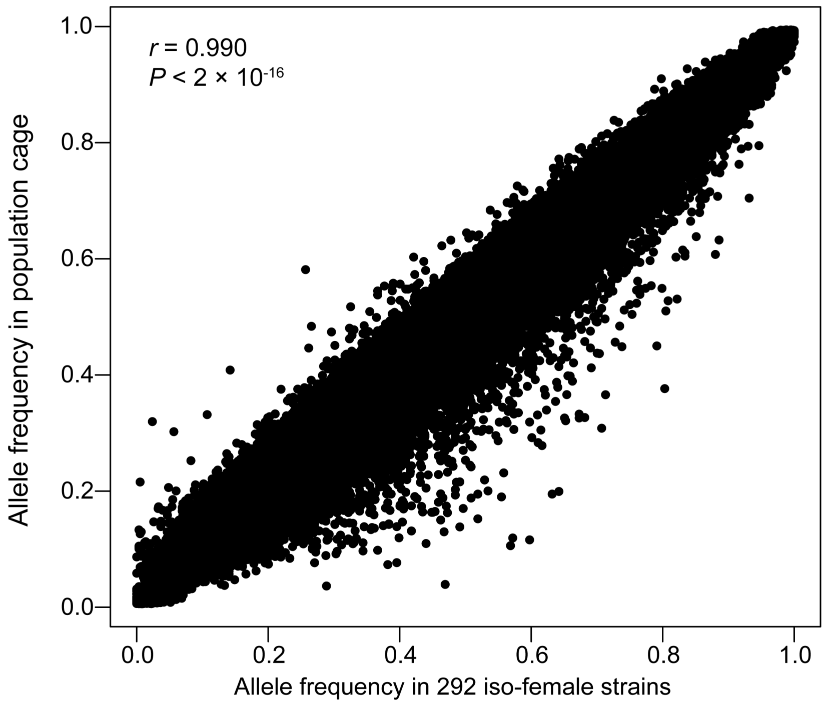

Fig. S2. The correlation between the allele frequency estimated from population cage Pool-seq samples and whole genome resequencing of 292 iso-female strains in China. The correlation coefficient (*r*) and *P* value were calculated by Pearson correlation method. 100,000 randomly selected variants were shown. The sequencing data of the 292 iso-female strains were from a previous study (*39*).

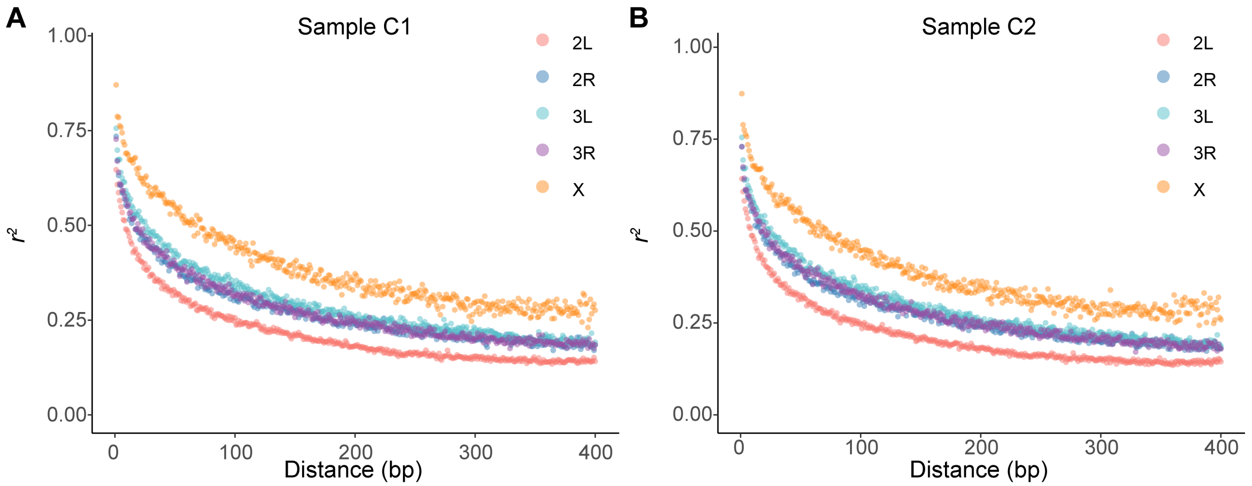

Fig. S3. Decay of linkage disequilibrium with physical distance for five major chromosome arms. The linkage disequilibrium (r^2) was estimated for pairwise SNPs (with a distance from 0–400 bp) across five major chromosome arms (2L, 2R, 3L, 3R, X) using LDx (*83*). The parameters include: minimum read depth 50× (-l 50), maximum depth 400× (-h 400), paired-end span 500 bp (-s 500), PHRED quality ≥ 20 (-q 20), and minor allele frequency ≥ 0.1 (-a 0.1).

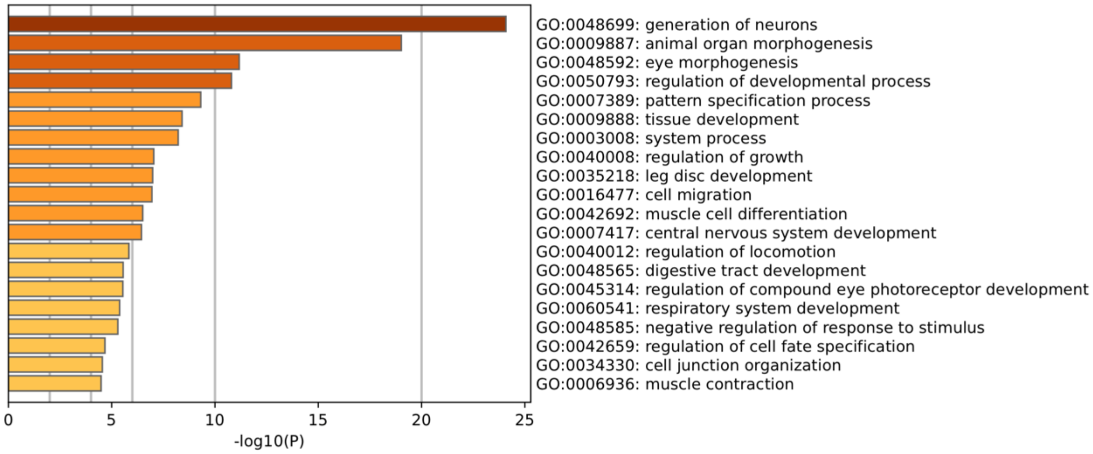

Fig. S4. Functional enrichment analysis of top 500 genes with the strongest associated variants. The enrichment analysis was performed by Metascape, focusing on biological processes and KEGG pathways. Genes harboring SNPs in the PCSS data were included in the background gene set. Enrichment criteria included: 1) a minimum of 3 overlapping genes, 2) P value < 0.01, and 3) a minimum fold enrichment of 1.5. The top 20 most enriched biological processes with P < 0.01 are shown.

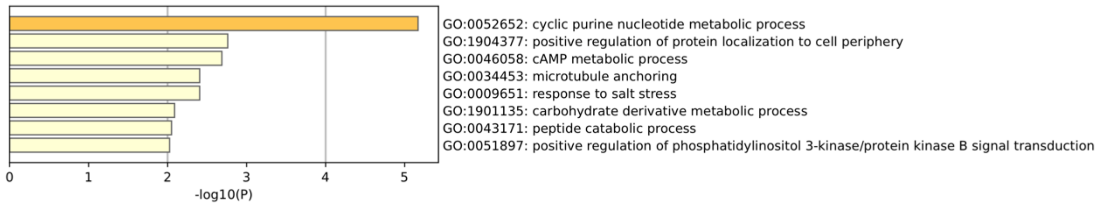

Fig. S5. Functional enrichment analysis of top 500 genes with candidate missense variants. The enrichment analysis was performed by Metascape, focusing on biological processes and KEGG pathways. All genes with polymorphic loci were used as the background. The enriched biological processes (eight GO terms) with $P < 0.01$ are shown.

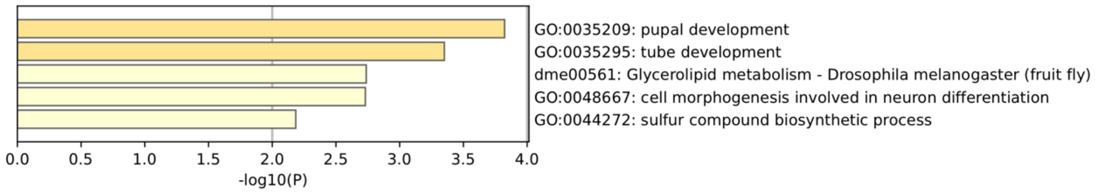

Fig. S6. Functional enrichment analysis of 79 genes with uORF variants. The enrichment analysis was performed by Metascape, focusing on biological processes and KEGG pathways. All genes with polymorphic loci were used as the background. The enriched biological processes (four GO terms) and one enriched KEGG pathway with $P < 0.01$ are shown.

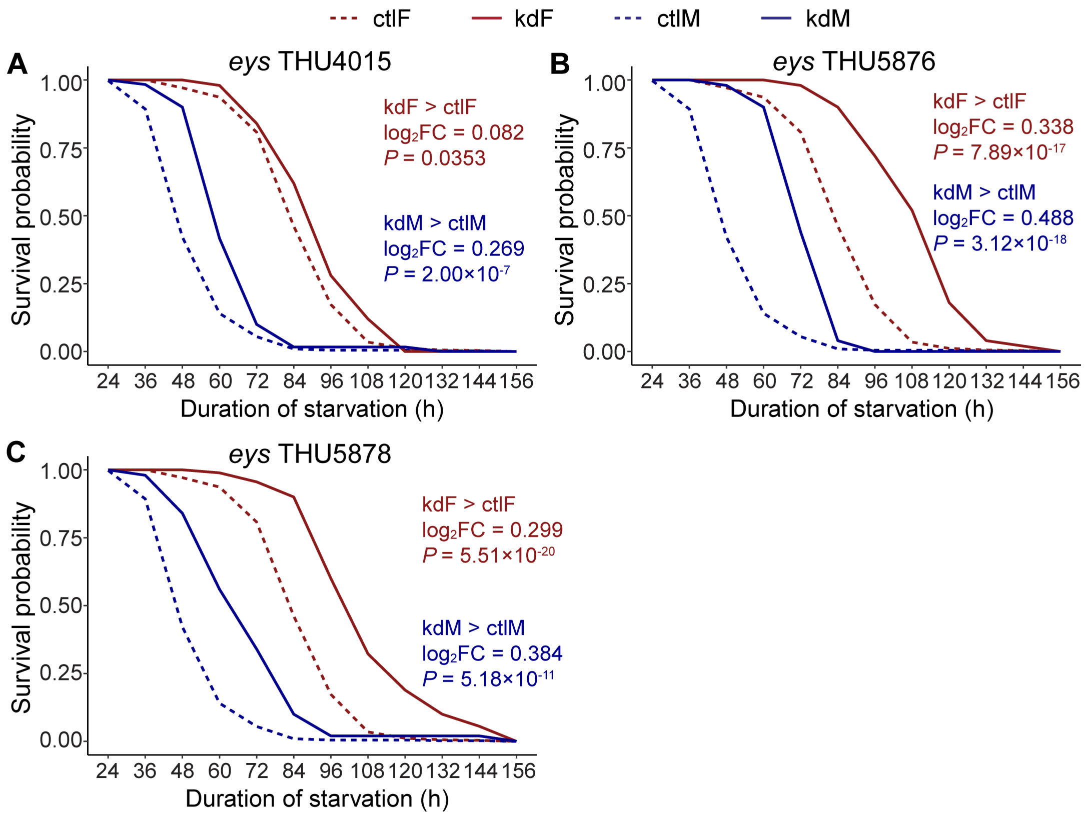

Fig. S7. RNAi of genes targeted by multiple strains. Starvation survival of multiple RNAi strains targeting *eys*. Knockdown (solid lines) and control (dashed lines) survival curves are shown for females (kdF, ctlF) and males (kdM, ctlM); $n \geq 50$ per genotype and sex. The $\log_2$FC of mean survival time (knockdown vs control; positive values indicate increased survival) is indicated in each panel. *P* values are from log-rank tests comparing knockdowns to controls within sex.

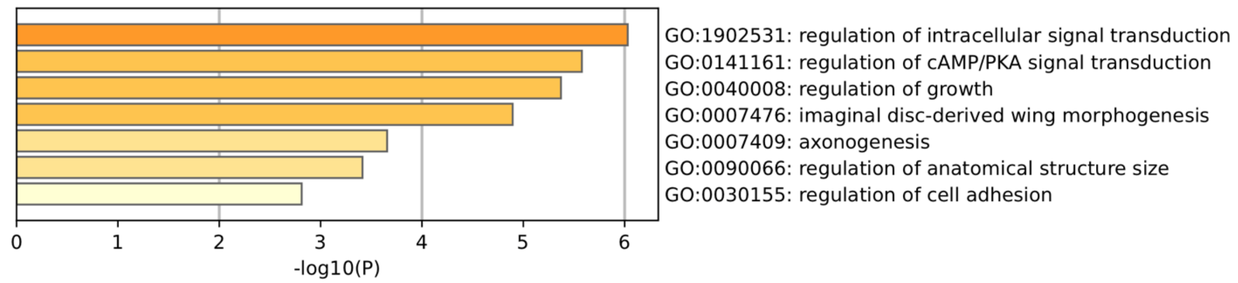

Fig. S8. Functional enrichment analysis of the 49 genes with sex-biased or sex antagonistic effects. The enrichment analysis was performed by Metascape, focusing on biological processes and KEGG pathways. All genes with polymorphic loci were used as the background. The enriched biological processes (seven GO terms) with $P < 0.01$ are shown.

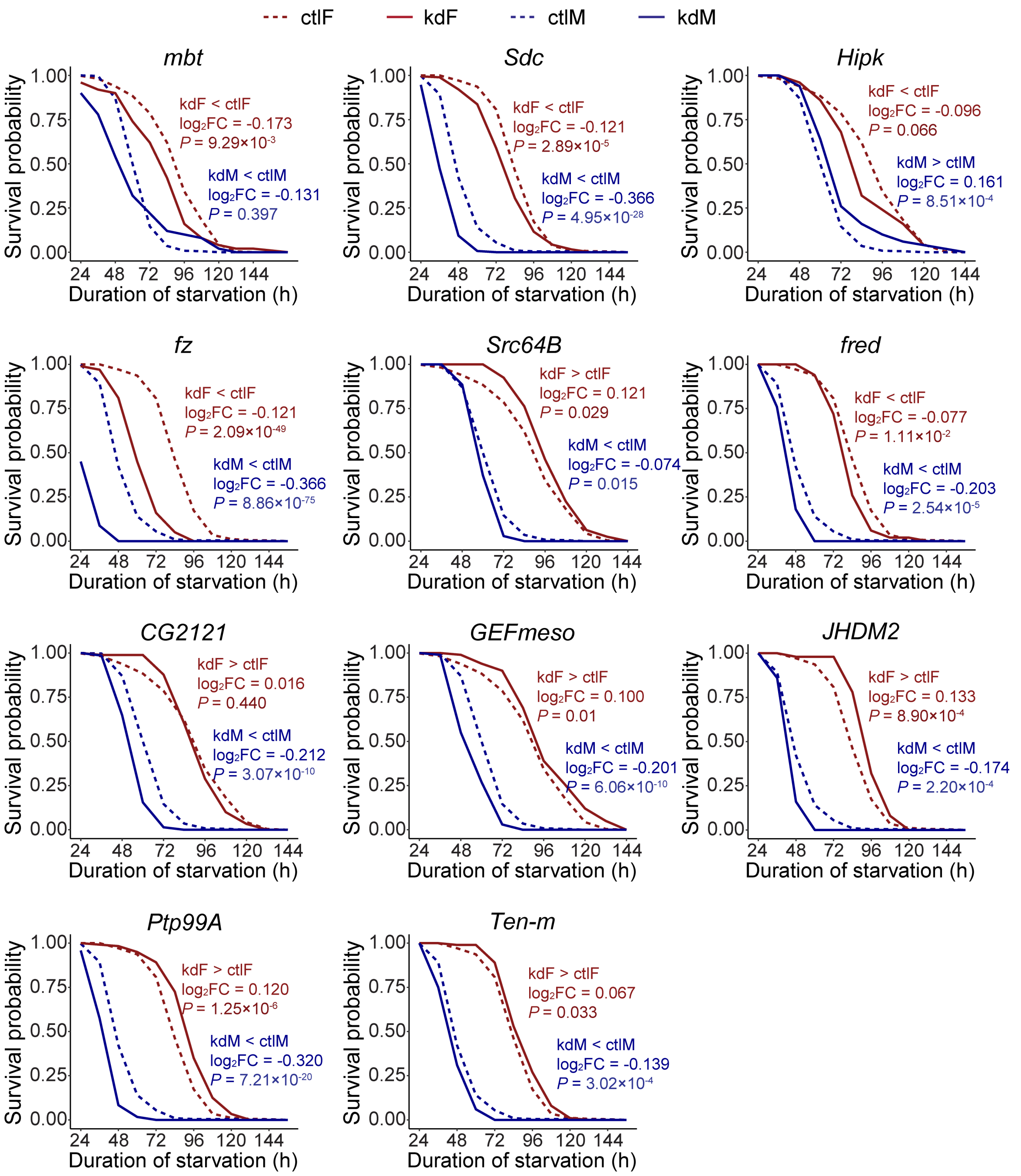

Fig. S9. Additional RNAi results for genes related to growth and Hippo signaling pathway, and genes with sexually antagonistic effects. RNAi results for genes functionally related to regulation of growth and the Hippo signaling pathway (*mbt*, *Sdc*, *Hipk*, *fz*, *Src64B*, *fred*, *CG2121*, and *Ten-m*) and genes with sexually antagonistic effects (*Src64B*, *GEFmeso*, *JHDM2*, *Ptp99A*, and *Ten-m*) are shown. Knockdown (solid lines) and control (dashed lines) survival curves are shown for females (kdF, ctlF) and males (kdM, ctlM); $n \geq 50$ per genotype and sex. The log_2FC of mean survival time (knockdown vs control; positive values indicate increased survival) is indicated in each panel. *P* values are from log-rank tests comparing knockdowns to controls within sex.

Table S1. Strains used in population cage experiment.

This table presents comprehensive details of the 200 iso-female strains used in the population cage experiment, containing information such as strain ID, strain tag, collection site, collection date, collector, altitude, latitude, and longitude.

Table S2. Phenotype sorting in the population cage experiment.

This table summarizes the sample collection process in the population cage experiment, including duration under starvation treatment, number of dead individuals collected at each time point from both cage replicates, and sample ID assigned.

Duration of starvation (hours)	Number of dead individuals (replicate A)	Number of dead individuals (replicate B)	Sample
0	37	58	
24	68	30	Sample1
36	46	52	Sample1
42	90	110	Sample1
48	118	174	Sample2
60	456	428	Sample3
66	313	367	Sample4
72	358	200	Sample5
84	203	261	Sample6
90	70	93	Sample7
96	27	28	Sample7
108	27	15	Sample7
114	5	4	Sample7
120	2	6	Sample7

Table S3. Number of variants detected by each sample.

This table presents the number and types of polymorphic sites identified in each pooled sequencing sample.

Sample	3′ UTR	5′ UTR	Intergenic	Intron	Missense	Synony-mous	Stop gained	Other types	Total
C1	55882	37669	426312	645287	51783	164167	214	27831	1409145
C2	55899	37685	426279	645226	51771	164160	214	27832	1409066
A1	55243	37266	421160	637531	51137	163347	202	27551	1393437
A2	55025	37194	419698	635170	50939	163149	200	27478	1388853
A3	55068	37131	419243	634703	50833	162971	201	27410	1387560
A4	55290	37357	421624	638036	51201	163422	206	27568	1394704
A5	55175	37232	419950	635588	50909	163207	207	27486	1389754
A6	55288	37342	421760	638320	51182	163461	200	27558	1395111
A7	54902	37159	419449	634775	51003	163204	201	27481	1388174
B1	55219	37293	421315	637694	51100	163305	200	27546	1393672
B2	55215	37281	420500	636634	51062	163328	201	27534	1391755
B3	55422	37342	422357	638814	51046	163359	199	27600	1396139
B4	55370	37378	421956	638444	51214	163528	209	27592	1395691
B5	55152	37189	419059	634985	50891	163190	200	27444	1388110
B6	55259	37325	421196	637451	51125	163426	207	27557	1393546
B7	55090	37142	420220	635840	50948	163014	196	27485	1389935

Table S4. Enrichment analysis of variant types.

This table details the enrichment analysis of starvation-tolerance-associated SNPs identified by the PCSS method across functional categories. Analyses were performed separately for all candidate SNPs, autosomal candidate SNPs, and X-chromosomal candidate SNPs. Results include enrichment score (log_2FC) and adjusted *P* values (Fisher's exact test with FDR correction).

Variant type	Type	Total	Type candidate	Total candidate	log_2FC	*P* (Fisher exact test)	FDR
Whole genome							
3′ UTR	51571	1285620	2296	57433	-4.92E-03	8.79E-01	8.79E-01
5′ UTR	35867	1285620	1679	57433	6.75E-02	5.89E-02	8.84E-02
intron	587234	1285620	25889	57433	-1.91E-02	4.72E-03	8.50E-03
missense	46753	1285620	2463	57433	2.38E-01	2.06E-15	6.19E-15
synonymous	153654	1285620	7827	57433	1.89E-01	1.53E-32	1.37E-31
intergenic	384358	1285620	16063	57433	-9.62E-02	2.78E-23	1.25E-22
uORF	2029	1285620	87	57433	-5.92E-02	7.47E-01	8.40E-01
miRNA target sites	17073	1285620	801	57433	7.07E-02	1.74E-01	2.24E-01
TF binding sites	516228	1285620	23619	57433	3.45E-02	3.62E-06	8.14E-06
Autosomes							
3′ UTR	45478	1159543	2091	52880	1.18E-02	7.06E-01	7.94E-01
5′ UTR	32168	1159543	1578	52880	1.05E-01	4.53E-03	8.15E-03
intron	524212	1159543	23635	52880	-1.65E-02	2.07E-02	3.10E-02
missense	42298	1159543	2288	52880	2.46E-01	2.62E-15	7.87E-15
synonymous	137147	1159543	7088	52880	1.80E-01	4.66E-27	4.20E-26
intergenic	354038	1159543	15065	52880	-9.99E-02	9.99E-24	4.50E-23
uORF	1825	1159543	83	52880	-3.95E-03	1.00E+00	1.00E+00
miRNA target sites	15107	1159543	742	52880	1.07E-01	4.80E-02	6.17E-02
TF binding sites	468890	1159543	21846	52880	3.09E-02	6.22E-05	1.40E-04
X chromosome							
3′ UTR	6093	125926	205	4551	-1.03E-01	3.24E-01	3.81E-01
5′ UTR	3699	125926	101	4551	-4.04E-01	4.04E-03	1.21E-02
intron	63008	125926	2253	4551	-1.54E-02	4.88E-01	4.88E-01
missense	4452	125926	175	4551	1.21E-01	2.71E-01	3.81E-01
synonymous	16507	125926	739	4551	3.09E-01	2.61E-09	2.35E-08
intergenic	30216	125926	997	4551	-1.31E-01	1.14E-03	5.11E-03
uORF	204	125926	4	4551	-8.82E-01	3.39E-01	3.81E-01
miRNA target sites	1966	125926	59	4551	-2.68E-01	1.79E-01	3.22E-01
TF binding sites	47338	125926	1773	4551	5.15E-02	6.17E-02	1.39E-01

Table S5. Enrichment analysis of regulatory tracks annotated by modENCODE.

This table presents the enrichment analysis of starvation-tolerance-associated SNPs identified by PCSS within regulatory elements, including transcription factor binding sites (TFBSs) and histone modification regions annotated by modENCODE. Results display enrichment score (log_2FC) and adjusted *P* values (Fisher's exact test with FDR correction).

Table S6. Frequency of *Lnk V58M* in natural populations.

This table presents the allele frequency of the *V58M* mutation in the *Lnk* gene across natural populations, along with their geographic origins. Population data were obtained from a previously published study (*39*).

Population	Frequency of *V58M*	Latitude (°)	Longitude (°)	Region
B	0.107	39.91	116.41	Asia
CN_QTP	0.048	29.65	91.18	Asia
CN_XJ	0.240	43.89	87.55	Asia
FR	0.286	45.77	4.86	Europe
N	0.158	52.02	5.1	Europe
STO	0.423	59.34	17.94	Europe
CAS	0.219	40.9	17.16	Europe
EG	0.186	30.1	31.32	Northern Africa
T	0.250	-42.77	147.56	Oceania
I	0.289	42.35	-76.57	North America
RAL	0.211	35.76	-78.66	North America
US_GA	0.267	33.96	-83.37	North America
EA	0.000	8.25	34.59	sub-Saharan Africa
EF	0.051	9.81	38.63	sub-Saharan Africa
CO	0.250	6.25	10.43	sub-Saharan Africa
GH	0.133	5.55	-0.2	sub-Saharan Africa
RG	0.080	-2.49	28.92	sub-Saharan Africa
SD	0.278	-25.42	30.1	sub-Saharan Africa
SP	0.237	-23.94	31.14	sub-Saharan Africa
KF	0.000	-14.69	26.39	sub-Saharan Africa
ZI	0.048	-16.54	28.72	sub-Saharan Africa

Table S7. The ANOVA of starvation resistance phenotype of *Lnk* transgenic strains.

This table presents the results of the ANOVA for starvation tolerance phenotypes of *Lnk* allele replacement lines (*Lnk-V* and *Lnk-M*). The factors in the model include genotype, sex, and their interaction term.

Source	Df	Sum_Sq	Mean_Sq	F_value	Pr (> F)
Genotype	1	26630	26630	161.16	<2.00E-16
Sex	1	331174	331174	2004.13	<2.00E-16
Genotype:Sex	1	6479	6479	39.21	5.05E-10
Residuals	1394	230353	165		

Table S8. RNAi results of candidate genes.

This table details the RNAi knockdown experiments, including RNAi strain IDs, target gene names, phenotypic changes after RNAi knockdown, *P* value for the genotype-gender interaction (calculated using coxph model), and sex-biased gene expression. The gene expression data for male and female adults were sourced separately from modENCODE. The genes are classified as male-biased and female-biased based on the female-to-male (RPKM) ratio (female-biased: ratio > 2, male-biased: ratio < 0.5).

Table S9. RNAi knockdown efficiency across sexes.

This table presents qPCR-measured target gene expression levels in F1 adults from 49 RNAi strains × Ubi-Gal4 driver strain. The relative expression levels in females and males after knockdown (normalized to the controls' expression) are provided. The experiment has 3 biological replicates and 2 technical replicates per group. *P* values and adjusted *P* values (FDR) were obtained from *t*-tests for knockdown efficiency differences across sex.

Table S10. Sex-biased gene expression and phenotypic effect.

This table presents the correlation between sex-biased expression patterns of starvation-tolerance candidate genes and sex-dependent phenotypic impact patterns after RNAi knockdown. Statistical significance was assessed using the Fisher-Freeman-Halton test.

Group	Female-biased expression	Male-biased expression	No bias	*P* (Fisher-Freeman-Halton test)*
Antagonistic effect	1	4	7	0.763
Consistent effect	2	7	8	0.816
Female-biased effect	1	3	1	0.356
Male-biased effect	6	9	17	0.574
No effect	2	4	5	1
Total	12	27	38	

* The exact *P* value is obtained through 10,000 simulations.

Table S11. sgRNAs used for reconstructing *Lnk* V58M transgenic strains.

sgRNA name	Sequence
Lnk-sgRNA-F	5′ TTCGTGCATACGAAGACACTCCCA 3′
Lnk-sgRNA-R	5′ AAACTGGGAGTGTCTTCGTATGCA 3′

Table S12. Primers used for qPCR.

REFERENCES AND NOTES

1. N. A. Karp, J. Mason, A. L. Beaudet, Y. Benjamini, L. Bower, R. E. Braun, S. D. M. Brown, E. J. Chesler, M. E. Dickinson, A. M. Flenniken, H. Fuchs, M. H. Angelis, X. Gao, S. Guo, S. Greenaway, R. Heller, Y. Herault, M. J. Justice, N. Kurbatova, C. J. Lelliott, K. C. K. Lloyd, A. M. Mallon, J. E. Mank, H. Masuya, C. McKerlie, T. F. Meehan, R. F. Mott, S. A. Murray, H. Parkinson, R. Ramirez-Solis, L. Santos, J. R. Seavitt, D. Smedley, T. Sorg, A. O. Speak, K. P. Steel, K. L. Svenson, S. Wakana, D. West, S. Wells, H. Westerberg, S. Yaacoby, J. K. White, Prevalence of sexual dimorphism in mammalian phenotypic traits. *Nat. Commun.* **8**, 15475 (2017).

2. R. Bonduriansky, S. F. Chenoweth, Intralocus sexual conflict. *Trends Ecol. Evol.* **24**, 280–288 (2009).

3. W. R. Rice, A. K. Chippindale, Intersexual ontogenetic conflict. *J. Evol. Biol.* **14**, 685–693 (2001).

4. G. Arnqvist, L. Rowe, *Sexual conflict* (Princeton Univ. Press, 2005), vol. **27**, 10.1515/9781400850600.

5. D. J. Fairbairn, *Odd Couples: Extraordinary Differences between the Sexes in the Animal Kingdom* (Princeton Univ. Press, 2013), 10.1515/9781400847600.

6. T. Connallon, A. G. Clark, Evolutionary inevitability of sexual antagonism. *Proc. Biol. Sci.* **281**, 20132123 (2014).

7. L. Rowe, S. F. Chenoweth, A. F. Agrawal, The genomics of sexual conflict. *Am. Nat.* **192**, 274–286 (2018).

8. T. M. Pennell, E. H. Morrow, Two sexes, one genome: The evolutionary dynamics of intralocus sexual conflict. *Ecol. Evol.* **3**, 1819–1834 (2013).

9. P. D. Price, S. M. Parkus, A. E. Wright, Recent progress in understanding the genomic architecture of sexual conflict. *Curr. Opin. Genet. Dev.* **80**, 102047 (2023).

10. N. M. Tosto, E. R. Beasley, B. B. M. Wong, J. E. Mank, S. P. Flanagan, The roles of sexual selection and sexual conflict in shaping patterns of genome and transcriptome variation. *Nat. Ecol. Evol.* **7**, 981–993 (2023).

11. S. C. Stearns, *The Evolution of Life Histories* (Oxford Univ. Press, 1998), 10.1093/oso/9780198577416.001.0001.

12. E. Gefen, A. G. Gibbs, Interactions between environmental stress and male mating success may enhance evolutionary divergence of stress-resistant *Drosophila* populations. *Evolution* **63**, 1653–1659 (2009).

13. S. P. De Lisle, D. Goedert, A. M. Reedy, E. I. Svensson, Climatic factors and species range position predict sexually antagonistic selection across taxa. *Philos. Trans. R. Soc. Lond. B Biol. Sci.* **373**, 20170415 (2018).

14. D. Berger, K. Grieshop, M. I. Lind, J. Goenaga, A. A. Maklakov, G. Arnqvist, Intralocus sexual conflict and environmental stress. *Evolution* **68**, 2184–2196 (2014).

15. T. Connallon, M. D. Hall, Genetic correlations and sex-specific adaptation in changing environments. *Evolution* **70**, 2186–2198 (2016).

16. A. D. Stewart, W. R. Rice, Arrest of sex-specific adaptation during the evolution of sexual dimorphism in *Drosophila*. *Nat. Ecol. Evol.* **2**, 1507–1513 (2018).

17. Tristan A. F. Long, Aneil F. Agrawal, L. Rowe, The effect of sexual selection on offspring fitness depends on the nature of genetic variation. *Curr. Biol.* **22**, 204–208 (2012).

18. F. Ruzicka, M. S. Hill, T. M. Pennell, I. Flis, F. C. Ingleby, R. Mott, K. Fowler, E. H. Morrow, M. Reuter, Genome-wide sexually antagonistic variants reveal long-standing constraints on sexual dimorphism in fruit flies. *PLOS Biol.* **17**, e3000244 (2019).

19. A. Sayadi, A. Martinez Barrio, E. Immonen, J. Dainat, D. Berger, C. Tellgren-Roth, B. Nystedt, G. Arnqvist, The genomic footprint of sexual conflict. *Nat. Ecol. Evol.* **3**, 1725–1730 (2019).

20. T. Connallon, A. G. Clark, Balancing selection in species with separate sexes: Insights from Fisher's geometric model. *Genetics* **197**, 991–1006 (2014).

21. F. Ruzicka, L. Dutoit, P. Czuppon, C. Y. Jordan, X. Y. Li, C. Olito, A. Runemark, E. I. Svensson, H. P. Yazdi, T. Connallon, The search for sexually antagonistic genes: Practical insights from studies of local adaptation and statistical genomics. *Evol. Lett.* **4**, 398–415 (2020).

22. J. Sun, C. Liu, X. Bai, X. Li, J. Li, Z. Zhang, Y. Zhang, J. Guo, Y. Li, *Drosophila* FIT is a protein-specific satiety hormone essential for feeding control. *Nat. Commun.* **8**, 14161 (2017).

23. C. J. Scheitz, Y. Guo, A. M. Early, L. G. Harshman, A. G. Clark, Heritability and inter-population differences in lipid profiles of *Drosophila melanogaster*. *PLOS ONE* **8**, e72726 (2013).

24. A. K. Chippindale, J. R. Gibson, W. R. Rice, Negative genetic correlation for adult fitness between sexes reveals ontogenetic conflict in *Drosophila*. *Proc. Natl. Acad. Sci. U.S.A.* **98**, 1671–1675 (2001).

25. L. Kwan, S. Bedhomme, N. G. Prasad, A. K. Chippindale, Sexual conflict and environmental change: Trade-offs within and between the sexes during the evolution of desiccation resistance. *J. Genet.* **87**, 383–394 (2008).

26. N. W. VanKuren, M. Long, Gene duplicates resolving sexual conflict rapidly evolved essential gametogenesis functions. *Nat. Ecol. Evol.* **2**, 705–712 (2018).

27. A. Glaser-Schmitt, M. J. Wittmann, T. J. S. Ramnarine, J. Parsch, Sexual antagonism, temporally fluctuating selection, and variable dominance affect a regulatory polymorphism in *Drosophila melanogaster*. *Mol. Biol. Evol.* **38**, 4891–4907 (2021).

28. W. G. Rostant, C. Kay, N. Wedell, D. J. Hosken, Sexual conflict maintains variation at an insecticide resistance locus. *BMC Biol.* **13**, 34 (2015).

29. S. T. Harbison, A. H. Yamamoto, J. J. Fanara, K. K. Norga, T. F. Mackay, Quantitative trait loci affecting starvation resistance in *Drosophila melanogaster*. *Genetics* **166**, 1807–1823 (2004).

30. E. R. Everman, C. L. McNeil, J. L. Hackett, C. L. Bain, S. J. Macdonald, Dissection of complex, fitness-related traits in multiple *Drosophila* mapping populations offers insight into the genetic control of stress resistance. *Genetics* **211**, 1449–1467 (2019).

31. T. F. Mackay, S. Richards, E. A. Stone, A. Barbadilla, J. F. Ayroles, D. Zhu, S. Casillas, Y. Han, M. M. Magwire, J. M. Cridland, M. F. Richardson, R. R. Anholt, M. Barrón, C. Bess, K. P. Blankenburg, M. A. Carbone, D. Castellano, L. Chaboub, L. Duncan, Z. Harris, M. Javaid, J. C. Jayaseelan, S. N. Jhangiani, K. W. Jordan, F. Lara, F. Lawrence, S. L. Lee, P. Librado, R. S. Linheiro, R. F. Lyman, A. J. Mackey, M. Munidasa, D. M. Muzny, L. Nazareth, I. Newsham, L. Perales, L. L. Pu, C. Qu, M. Ràmia, J. G. Reid, S. M. Rollmann, J. Rozas, N. Saada, L. Turlapati, K. C. Worley, Y. Q. Wu, A. Yamamoto, Y. Zhu, C. M. Bergman, K. R. Thornton, D. Mittelman, R. A. Gibbs, The *Drosophila melanogaster* genetic reference panel. *Nature* **482**, 173–178 (2012).

32. W. Huang, A. Massouras, Y. Inoue, J. Peiffer, M. Ramia, A. M. Tarone, L. Turlapati, T. Zichner, D. Zhu, R. F. Lyman, M. M. Magwire, K. Blankenburg, M. A. Carbone, K. Chang, L. L. Ellis, S. Fernandez, Y. Han, G. Highnam, C. E. Hjelmen, J. R. Jack, M. Javaid, J. Jayaseelan, D. Kalra, S. Lee, L. Lewis, M. Munidasa, F. Ongeri, S. Patel, L. Perales, A. Perez, L. Pu, S. M. Rollmann, R. Ruth, N. Saada, C. Warner, A. Williams, Y. Q. Wu, A. Yamamoto, Y. Zhang, Y. Zhu, R. R. Anholt, J. O. Korbel, D. Mittelman, D. M. Muzny, R. A. Gibbs, A. Barbadilla, J. S. Johnston, E. A. Stone, S. Richards, B. Deplancke, T. F. Mackay, Natural variation in genome architecture among 205 *Drosophila melanogaster* genetic reference panel lines. *Genome Res.* **24**, 1193–1208 (2014).

33. C. M. Hardy, M. K. Burke, L. J. Everett, M. V. Han, K. M. Lantz, A. G. Gibbs, Genome-wide analysis of starvation-selected *Drosophila melanogaster*—A genetic model of obesity. *Mol. Biol. Evol.* **35**, 50–65 (2018).

34. P. Michalak, L. Kang, M. F. Schou, H. R. Garner, V. Loeschcke, Genomic signatures of experimental adaptive radiation in *Drosophila*. *Mol. Ecol.* **28**, 600–614 (2019).

35. J. G. Sorensen, M. M. Nielsen, V. Loeschcke, Gene expression profile analysis of *Drosophila melanogaster* selected for resistance to environmental stressors. *J. Evol. Biol.* **20**, 1624–1636 (2007).

36. S. M. Rudman, S. I. Greenblum, S. Rajpurohit, N. J. Betancourt, J. Hanna, S. Tilk, T. Yokoyama, D. A. Petrov, P. Schmidt, Direct observation of adaptive tracking on ecological time scales in *Drosophila*. *Science* **375**, eabj7484 (2022).

37. D. P. Sarikaya, J. Cridland, A. Tarakji, H. Sheehy, S. Davis, A. Kochummen, R. Hatmaker, N. Khan, J. Chiu, D. J. Begun, Phenotypic coupling of sleep and starvation resistance evolves in *D. melanogaster*. *BMC Evol. Biol.* **20**, 126 (2020).

38. R. Kofler, P. Orozco-terWengel, N. De Maio, R. V. Pandey, V. Nolte, A. Futschik, C. Kosiol, C. Schlotterer, PoPoolation: A toolbox for population genetic analysis of next generation sequencing data from pooled individuals. *PLOS ONE* **6**, e15925 (2011).

39. J. Chen, C. Liu, W. Li, W. Zhang, Y. Wang, A. G. Clark, J. Lu, From sub-Saharan Africa to China: Evolutionary history and adaptation of *Drosophila melanogaster* revealed by population genomics. *Sci. Adv.* **10**, eadh3425 (2024).

40. T. Glatter, R. B. Schittenhelm, O. Rinner, K. Roguska, A. Wepf, M. A. Junger, K. Kohler, I. Jevtov, H. Choi, A. Schmidt, A. I. Nesvizhskii, H. Stocker, E. Hafen, R. Aebersold, M. Gstaiger, Modularity and hormone sensitivity of the *Drosophila melanogaster* insulin receptor/target of rapamycin interaction proteome. *Mol. Syst. Biol.* **7**, 547 (2011).

41. G. Tettweiler, M. Miron, M. Jenkins, N. Sonenberg, P. F. Lasko, Starvation and oxidative stress resistance in *Drosophila* are mediated through the eIF4E-binding protein, d4E-BP. *Genes Dev.* **19**, 1840–1843 (2005).

42. modENCODE Consortium, S. Roy, J. Ernst, P. V. Kharchenko, P. Kheradpour, N. Negre, M. L. Eaton, J. M. Landolin, C. A. Bristow, L. Ma, M. F. Lin, S. Washietl, B. I. Arshinoff, F. Ay, P. E. Meyer, N. Robine, N. L. Washington, L. Di Stefano, E. Berezikov, C. D. Brown, R. Candeias, J. W. Carlson, A. Carr, I. Jungreis, D. Marbach, R. Sealfon, M. Y. Tolstorukov, S. Will, A. A.

Alekseyenko, C. Artieri, B. W. Booth, A. N. Brooks, Q. Dai, C. A. Davis, M. O. Duff, X. Feng, A. A. Gorchakov, T. Gu, J. G. Henikoff, P. Kapranov, R. Li, H. K. MacAlpine, J. Malone, A. Minoda, J. Nordman, K. Okamura, M. Perry, S. K. Powell, N. C. Riddle, A. Sakai, A. Samsonova, J. E. Sandler, Y. B. Schwartz, N. Sher, R. Spokony, D. Sturgill, M. van Baren, K. H. Wan, L. Yang, C. Yu, E. Feingold, P. Good, M. Guyer, R. Lowdon, K. Ahmad, J. Andrews, B. Berger, S. E. Brenner, M. R. Brent, L. Cherbas, S. C. Elgin, T. R. Gingeras, R. Grossman, R. A. Hoskins, T. C. Kaufman, W. Kent, M. I. Kuroda, T. Orr-Weaver, N. Perrimon, V. Pirrotta, J. W. Posakony, B. Ren, S. Russell, P. Cherbas, B. R. Graveley, S. Lewis, G. Micklem, B. Oliver, P. J. Park, S. E. Celniker, S. Henikoff, G. H. Karpen, E. C. Lai, D. M. MacAlpine, L. D. Stein, K. P. White, M. Kellis, Identification of functional elements and regulatory circuits by *Drosophila* modENCODE. *Science* **330**, 1787–1797 (2010).

43. M. Pandey, S. Bansal, S. Bar, A. K. Yadav, N. S. Sokol, J. M. Tennessen, P. Kapahi, G. Chawla, *miR-125-chinmo* pathway regulates dietary restriction-dependent enhancement of lifespan in *Drosophila*. *eLife* **10**, e62621 (2021).

44. H. Zhang, Y. Wang, J. Lu, Function and evolution of upstream ORFs in eukaryotes. *Trends Biochem. Sci.* **44**, 782–794 (2019).

45. C. Slack, C. Werz, D. Wieser, N. Alic, A. Foley, H. Stocker, D. J. Withers, J. M. Thornton, E. Hafen, L. Partridge, Regulation of lifespan, metabolism, and stress responses by the *Drosophila* SH2B protein, Lnk. *PLoS Genet.* **6**, e1000881 (2010).

46. W. Song, D. Ren, W. Li, L. Jiang, K. W. Cho, P. Huang, C. Fan, Y. Song, Y. Liu, L. Rui, SH2B regulation of growth, metabolism, and longevity in both insects and mammals. *Cell Metab.* **11**, 427–437 (2010).

47. F.-X. Yu, B. Zhao, K.-L. Guan, Hippo pathway in organ size control, tissue homeostasis, and cancer. *Cell* **163**, 811–828 (2015).

48. T. Connallon, R. M. Cox, R. Calsbeek, Fitness consequences of sex-specific selection. *Evolution* **64**, 1671–1682 (2010).

49. F. C. Ingleby, I. Flis, E. H. Morrow, Sex-biased gene expression and sexual conflict throughout development. *Cold Spring Harb. Perspect. Biol.* **7**, a017632 (2014).

50. M. A. Pointer, P. W. Harrison, A. E. Wright, J. E. Mank, Masculinization of gene expression is associated with exaggeration of male sexual dimorphism. *PLOS Genet.* **9**, e1003697 (2013).

51. J. Parsch, H. Ellegren, The evolutionary causes and consequences of sex-biased gene expression. *Nat. Rev. Genet.* **14**, 83–87 (2013).

52. P. Innocenti, E. H. Morrow, The sexually antagonistic genes of *Drosophila melanogaster*. *PLOS Biol.* **8**, e1000335 (2010).

53. C. Cheng, M. Kirkpatrick, Sex-specific selection and sex-biased gene expression in humans and flies. *PLOS Genet.* **12**, e1006170 (2016).

54. J. M. Ranz, C. I. Castillo-Davis, C. D. Meiklejohn, D. L. Hartl, Sex-dependent gene expression and evolution of the *Drosophila* transcriptome. *Science* **300**, 1742–1745 (2003).

55. S. Rion, T. J. Kawecki, Evolutionary biology of starvation resistance: what we have learned from *Drosophila*. *J. Evol. Biol.* **20**, 1655–1664 (2007).

56. A. B. Paaby, M. J. Blacket, A. A. Hoffmann, P. S. Schmidt, Identification of a candidate adaptive polymorphism for *Drosophila* life history by parallel independent clines on two continents. *Mol. Ecol.* **19**, 760–774 (2010).

57. N. J. Betancourt, S. Rajpurohit, E. Durmaz, D. K. Fabian, M. Kapun, T. Flatt, P. Schmidt, Allelic polymorphism at *foxo* contributes to local adaptation in *Drosophila melanogaster*. *Mol. Ecol.* **30**, 2817–2830 (2021).

58. A. B. Paaby, A. O. Bergland, E. L. Behrman, P. S. Schmidt, A highly pleiotropic amino acid polymorphism in the *Drosophila* insulin receptor contributes to life-history adaptation. *Evolution* **68**, 3395–3409 (2014).

59. E. Durmaz, S. Rajpurohit, N. Betancourt, D. K. Fabian, M. Kapun, P. Schmidt, T. Flatt, A clinal polymorphism in the insulin signaling transcription factor *foxo* contributes to life-history adaptation in *Drosophila*. *Evolution* **73**, 1774–1792 (2019).

60. C. Bass, L. M. Field, Gene amplification and insecticide resistance. *Pest Manag. Sci.* **67**, 886–890 (2011).

61. J. S. Ayres, N. Freitag, D. S. Schneider, Identification of *Drosophila* mutants altering defense of and endurance to *Listeria monocytogenes* infection. *Genetics* **178**, 1807–1815 (2008).

62. T. J. Kawecki, B. Erkosar, C. Dupuis, B. Hollis, R. C. Stillwell, M. Kapun, The genomic architecture of adaptation to larval malnutrition points to a trade-off with adult starvation resistance in *Drosophila*. *Mol. Biol. Evol.* **38**, 2732–2749 (2021).

63. E. R. Everman, T. J. Morgan, Antagonistic pleiotropy and mutation accumulation contribute to age-related decline in stress response. *Evolution* **72**, 303–317 (2018).

64. E. A. Lucotte, R. Laurent, E. Heyer, L. Ségurel, B. Toupance, Detection of allelic frequency differences between the sexes in humans: A signature of sexually antagonistic selection. *Genome Biol. Evol.* **8**, 1489–1500 (2016).

65. J. C. Randall, T. W. Winkler, Z. Kutalik, S. I. Berndt, A. U. Jackson, K. L. Monda, T. O. Kilpeläinen, T. Esko, R. Mägi, S. Li, T. Workalemahu, M. F. Feitosa, D. C. Croteau-Chonka, F. R. Day, T. Fall, T. Ferreira, S. Gustafsson, A. E. Locke, I. Mathieson, A. Scherag, S. Vedantam, A. R. Wood, L. Liang, V. Steinthorsdottir, G. Thorleifsson, E. T. Dermitzakis, A. S. Dimas, F. Karpe, J. L. Min, G. Nicholson, D. J. Clegg, T. Person, J. P. Krohn, S. Bauer, C. Buechler, K. Eisinger, DIAGRAM Consortium, A. Bonnefond, P. Froguel, MAGIC Investigators, J. J. Hottenga, I. Prokopenko, L. L. Waite, T. B. Harris, A. V. Smith, A. R. Shuldiner, W. L. McArdle, M. J. Caulfield, P. B. Munroe, H. Grönberg, Y. D. Chen, G. Li, J. S. Beckmann, T. Johnson, U. Thorsteinsdottir, M. Teder-Laving, K. T. Khaw, N. J. Wareham, J. H. Zhao, N. Amin, B. A. Oostra, A. T. Kraja, M. A. Province, L. A. Cupples, N. L. Heard-Costa, J. Kaprio, S. Ripatti, I. Surakka, F. S. Collins, J. Saramies, J. Tuomilehto, A. Jula, V. Salomaa, J. Erdmann, C. Hengstenberg, C. Loley, H. Schunkert, C. Lamina, H. E. Wichmann, E. Albrecht, C. Gieger,

A. A. Hicks, A. Johansson, P. P. Pramstaller, S. Kathiresan, E. K. Speliotes, B. Penninx, A. L. Hartikainen, M. R. Jarvelin, U. Gyllensten, D. I. Boomsma, H. Campbell, J. F. Wilson, S. J. Chanock, M. Farrall, A. Goel, C. Medina-Gomez, F. Rivadeneira, K. Estrada, A. G. Uitterlinden, A. Hofman, M. C. Zillikens, M. den Heijer, L. A. Kiemeney, A. Maschio, P. Hall, J. Tyrer, A. Teumer, H. Völzke, P. Kovacs, A. Tönjes, M. Mangino, T. D. Spector, C. Hayward, I. Rudan, A. S. Hall, N. J. Samani, A. P. Attwood, J. G. Sambrook, J. Hung, L. J. Palmer, M. L. Lokki, J. Sinisalo, G. Boucher, H. Huikuri, M. Lorentzon, C. Ohlsson, N. Eklund, J. G. Eriksson, C. Barlassina, C. Rivolta, I. M. Nolte, H. Snieder, M. M. Van der Klauw, J. V. Van Vliet-Ostaptchouk, P. V. Gejman, J. Shi, K. B. Jacobs, Z. Wang, S. J. Bakker, I. Mateo Leach, G. Navis, P. van der Harst, N. G. Martin, S. E. Medland, G. W. Montgomery, J. Yang, D. I. Chasman, P. M. Ridker, L. M. Rose, T. Lehtimäki, O. Raitakari, D. Absher, C. Iribarren, H. Basart, K. G. Hovingh, E. Hyppönen, C. Power, D. Anderson, J. P. Beilby, J. Hui, J. Jolley, H. Sager, S. R. Bornstein, P. E. Schwarz, K. Kristiansson, M. Perola, J. Lindström, A. J. Swift, M. Uusitupa, M. Atalay, T. A. Lakka, R. Rauramaa, J. L. Bolton, G. Fowkes, R. M. Fraser, J. F. Price, K. Fischer, K. Krjutå Kov, A. Metspalu, E. Mihailov, C. Langenberg, J. Luan, K. K. Ong, P. S. Chines, S. M. Keinanen-Kiukaanniemi, T. E. Saaristo, S. Edkins, P. W. Franks, G. Hallmans, D. Shungin, A. D. Morris, C. N. Palmer, R. Erbel, S. Moebus, M. M. Nöthen, S. Pechlivanis, K. Hveem, N. Narisu, A. Hamsten, S. E. Humphries, R. J. Strawbridge, E. Tremoli, H. Grallert, B. Thorand, T. Illig, W. Koenig, M. Müller-Nurasyid, A. Peters, B. O. Boehm, M. E. Kleber, W. März, B. R. Winkelmann, J. Kuusisto, M. Laakso, D. Arveiler, G. Cesana, K. Kuulasmaa, J. Virtamo, J. W. Yarnell, D. Kuh, A. Wong, L. Lind, U. de Faire, B. Gigante, P. K. Magnusson, N. L. Pedersen, G. Dedoussis, M. Dimitriou, G. Kolovou, S. Kanoni, K. Stirrups, L. L. Bonnycastle, I. Njølstad, T. Wilsgaard, A. Ganna, E. Rehnberg, A. Hingorani, M. Kivimaki, M. Kumari, T. L. Assimes, I. Barroso, M. Boehnke, I. B. Borecki, P. Deloukas, C. S. Fox, T. Frayling, L. C. Groop, T. Haritunians, D. Hunter, E. Ingelsson, R. Kaplan, K. L. Mohlke, J. R. O'Connell, D. Schlessinger, D. P. Strachan, K. Stefansson, C. M. van Duijn, G. R. Abecasis, M. I. McCarthy, J. N. Hirschhorn, L. Qi, R. J. Loos, C. M. Lindgren, K. E. North, I. M. Heid, Sex-stratified genome-wide association studies including 270,000 individuals show sexual dimorphism in genetic loci for anthropometric traits. *PLOS Genet.* **9**, e1003500 (2013).

66. K. Rawlik, O. Canela-Xandri, A. Tenesa, Evidence for sex-specific genetic architectures across a spectrum of human complex traits. *Genome Biol.* **17**, 166 (2016).

67. J. A. Harper, T. Janicke, E. H. Morrow, Systematic review reveals multiple sexually antagonistic polymorphisms affecting human disease and complex traits. *Evolution* **75**, 3087–3097 (2021).

68. N. J. Barson, T. Aykanat, K. Hindar, M. Baranski, G. H. Bolstad, P. Fiske, C. Jacq, A. J. Jensen, S. E. Johnston, S. Karlsson, M. Kent, T. Moen, E. Niemelä, T. Nome, T. F. Næsje, P. Orell, A. Romakkaniemi, H. Sægrov, K. Urdal, J. Erkinaro, S. Lien, C. R. Primmer, Sex-dependent dominance at a single locus maintains variation in age at maturity in salmon. *Nature* **528**, 405–408 (2015).

69. T. Akagi, D. Charlesworth, Pleiotropic effects of sex-determining genes in the evolution of dioecy in two plant species. *Proc. Biol. Sci.* **286**, 20191805 (2019).

70. H. Li, Aligning sequence reads, clone sequences and assembly contigs with BWA-MEM. arXiv:1303.3997 (2013).

71. E. Garrison, G. Marth, Haplotype-based variant detection from short-read sequencing. arXiv:1207.3907 (2012).

72. P. Danecek, J. K. Bonfield, J. Liddle, J. Marshall, V. Ohan, M. O. Pollard, A. Whitwham, T. Keane, S. A. McCarthy, R. M. Davies, H. Li, Twelve years of SAMtools and BCFtools. *Gigascience* **10**, giab008 (2021).

73. E. Garrison, Z. N. Kronenberg, E. T. Dawson, B. S. Pedersen, P. Prins, A spectrum of free software tools for processing the VCF variant call format: vcflib, bio-vcf, cyvcf2, hts-nim and slivar. *PLOS Comput. Biol.* **18**, e1009123 (2022).

74. P. Cingolani, A. Platts, L. L. Wang, M. Coon, T. Nguyen, L. Wang, S. J. Land, X. Lu, D. M. Ruden, A program for annotating and predicting the effects of single nucleotide polymorphisms, SnpEff: SNPs in the genome of *Drosophila melanogaster* strain w1118; iso-2; iso-3. *Fly* **6**, 80–92 (2012).

75. D. R. Cox, Regression models and life-tables. *J. R. Stat. Soc. B: Stat. Methodol.* **34**, 187–202 (1972).

76. T. Therneau, A package for survival analysis in R. R package version 3.8-3 (2024); https://CRAN.R-project.org/package=survival.

77. X. Zhang, M. Wakeling, J. Ware, N. Whiffin, Annotating high-impact 5′untranslated region variants with the UTRannotator. *Bioinformatics* **37**, 1171–1173 (2021).

78. W. McLaren, L. Gil, S. E. Hunt, H. S. Riat, G. R. Ritchie, A. Thormann, P. Flicek, F. Cunningham, The Ensembl variant effect predictor. *Genome Biol.* **17**, 122 (2016).

79. V. Agarwal, A. O. Subtelny, P. Thiru, I. Ulitsky, D. P. Bartel, Predicting microRNA targeting efficacy in *Drosophila*. *Genome Biol.* **19**, 152 (2018).

80. Y. Zhou, B. Zhou, L. Pache, M. Chang, A. H. Khodabakhshi, O. Tanaseichuk, C. Benner, S. K. Chanda, Metascape provides a biologist-oriented resource for the analysis of systems-level datasets. *Nat. Commun.* **10**, 1523 (2019).

81. X. Ren, Z. Yang, J. Xu, J. Sun, D. Mao, Y. Hu, S. J. Yang, H. H. Qiao, X. Wang, Q. Hu, P. Deng, L. P. Liu, J. Y. Ji, J. B. Li, J. Q. Ni, Enhanced specificity and efficiency of the CRISPR/Cas9 system with optimized sgRNA parameters in *Drosophila*. *Cell Rep.* **9**, 1151–1162 (2014).

82. P. Peng, X. Wang, D. Shen, J. Sun, Y. Jia, R. G. Xu, L. F. Zhu, J. Q. Ni, CRISPR-Cas9 mediated genome editing in *Drosophila*. *Bio Protoc.* **9**, e3141 (2019).

83. A. F. Feder, D. A. Petrov, A. O. Bergland, LDx: Estimation of linkage disequilibrium from high-throughput pooled resequencing data. *PLOS ONE* **7**, e48588 (2012).
